# Comprehensive Profiling of Amino Acid Response Uncovers Unique Methionine-Deprived Response Dependent on Intact Creatine Biosynthesis

**DOI:** 10.1371/journal.pgen.1005158

**Published:** 2015-04-07

**Authors:** Xiaohu Tang, Melissa M. Keenan, Jianli Wu, Chih-An Lin, Laura Dubois, J. Will Thompson, Stephen J. Freedland, Susan K. Murphy, Jen-Tsan Chi

**Affiliations:** 1 Department of Molecular Genetics and Microbiology, Duke University Medical Center, Durham, North Carolina, United States of America; 2 Center for Genomic and Computational Biology, Duke University Medical Center, Durham, North Carolina, United States of America; 3 Duke Proteomics and Metabolomics Core Facility Duke University Medical Center, Durham, North Carolina, United States of America; 4 Department of Pharmacology and Cancer Biology Duke University Medical Center, Durham, North Carolina, United States of America; 5 Department of Surgery Duke University Medical Center, Durham, North Carolina, United States of America; University of Southern California, UNITED STATES

## Abstract

Besides being building blocks for protein synthesis, amino acids serve a wide variety of cellular functions, including acting as metabolic intermediates for ATP generation and for redox homeostasis. Upon amino acid deprivation, free uncharged tRNAs trigger GCN2-ATF4 to mediate the well-characterized transcriptional amino acid response (AAR). However, it is not clear whether the deprivation of different individual amino acids triggers identical or distinct AARs. Here, we characterized the global transcriptional response upon deprivation of one amino acid at a time. With the exception of glycine, which was not required for the proliferation of MCF7 cells, we found that the deprivation of most amino acids triggered a shared transcriptional response that included the activation of ATF4, p53 and TXNIP. However, there was also significant heterogeneity among different individual AARs. The most dramatic transcriptional response was triggered by methionine deprivation, which activated an extensive and unique response in different cell types. We uncovered that the specific methionine-deprived transcriptional response required creatine biosynthesis. This dependency on creatine biosynthesis was caused by the consumption of S-Adenosyl-L-methionine (SAM) during creatine biosynthesis that helps to deplete SAM under methionine deprivation and reduces histone methylations. As such, the simultaneous deprivation of methionine and sources of creatine biosynthesis (either arginine or glycine) abolished the reduction of histone methylation and the methionine-specific transcriptional response. Arginine-derived ornithine was also required for the complete induction of the methionine-deprived specific gene response. Collectively, our data identify a previously unknown set of heterogeneous amino acid responses and reveal a distinct methionine-deprived transcriptional response that results from the crosstalk of arginine, glycine and methionine metabolism via arginine/glycine-dependent creatine biosynthesis.

## Author Summary

In order for mammalian cells to live and function, amino acids are required for protein synthesis and the generation of metabolic intermediates. An imbalance or deficiency of amino acids often triggers an “amino acid response” (AAR) to allow cells to adapt to their environment. However, it remains unclear whether the deprivation of any single amino acid leads to similar or different changes compared to the global AAR response or to other single amino acid deficiencies. To answer this question, we removed each or all of the 15 amino acids found in media from cells and comprehensively profiled the resulting changes in their RNA expression. Strikingly, we found a unique and dramatic gene expression program that occurred only when cells were deprived of methionine, but not any other amino acid. We also found that these methionine-specific changes depended on changes in histone modifications and an intact creatine biosynthesis pathway. Methionine deprivation reduced the degree to which histone proteins were indirectly modified by methionine (histone methylation). Creatine biosynthesis consumed methionine’s derivate S-Adenosyl-L-methionine (SAM), contributing to the reduction of histone methylation and an increase in ornithine-mediated signaling. Since methionine restriction may have anti-aging and other medical uses, our findings provide insights that will lead toward a better understanding of the underlying effects of methionine restriction and eventually improve human health.

## Introduction

While amino acids are the building blocks of proteins, different amino acids also participate in a wide variety of biological processes. For example, amino acids supply carbon and nitrogen molecules for biosynthesis, feed substrates to maintain TCA cycle activity for ATP generation, and provide reducing equivalents to bolster anti-stress capacity for redox homeostasis. Therefore, all organisms have developed strategies to cope with metabolic stress and challenges posed by the deprivation of amino acids. In mammalian cells, there are at least two major adaptive mechanisms that sense and respond to fluctuations in amino acids levels. Mammalian target of rapamycin (mTOR) is a conserved Ser/Thr kinase that senses amino acid availability to regulate cell growth and autophagy. Another important sensor is the GCN2 (general control nonderepressible 2) kinase that regulates protein translation initiation in amino acid–starved cells by detecting uncharged tRNAs. These two kinases are highly conserved from yeast to mammalian cells and play major roles in the control of protein translation, transcriptional programs, and regulation of adaptive responses during amino acid starvation. One of the downstream effects of amino acid deprivation is the phosphorylation of Ser51 on the α-subunit of eukaryotic translation initiation factor (eIF) 2α by GCN2, which causes reduced rates of translation initiation and a general decline in protein synthesis. Besides GCN2, three additional eIF2a upstream kinases, including heme regulated initiation factor 2α kinase (HRI), protein kinase R (PKR) and protein kinase R like ER kinase (PERK), guard translation initiation in response to distinct kinds of stress in mammals. All four kinases have highly similar downstream components as they all phosphorylate eIF2α on Serine 51. While phosphorylated eIF2α generally suppresses protein synthesis, it also promotes the translation of select mRNA species that contain unique features in their 5’ untranslated regions, such as the activating transcription factor 4 (ATF4) [1]. ATF4 triggers a general AAR by inducing expression of a large number of target genes, including activating transcription factor 3 (*ATF3*), CEBP homologous protein (*CHOP*), and asparagine synthetase (*ASNS*). An amino acid response element (AARE) in the promoters of these genes allows for the coordinated transcriptional regulation [2].

While the network of transcriptional changes of an AAR have been extensively investigated [3], our understanding is still limited in several ways. First, are there additional, yet-uncharacterized pathways elicited as cells respond to amino acid deprivation? Second, to what degree are similar or distinct responses triggered by deprivation of different individual amino acids? Third, whether or how is the transcriptional response affected by the crosstalk of amino acid metabolism?

Among the twenty-two standard amino acids, nine are considered “essential” because the human body must obtain these from nutritional intake. For different types of cells, there also might be different dependencies based on their genetic makeup and metabolic flexibility [4] as well as the microenvironmental stresses of these cancer [5,6]. There has been much interest in identifying nutrient addictions of cancer cells with the hope for new therapeutic opportunities. For example, acute lymphocytic leukemia (ALL) cells are deficient in the asparagine pathway and require large amounts of exogenous asparagine. Therefore, asparaginase, through its ability to deplete extracellular asparagine, has become a cornerstone in the treatment of ALL. Glutamine addiction is found in basal-type breast cancer cells [7,8] and cancer cells with activated Myc and Ras [9,10]. Certain melanoma cells have a leucine addiction caused by defective adaptive autophagy [11]. An important implication of these studies is the significant heterogeneity in how cells sense and respond to the deprivation of each amino acid.

Although the cellular response to general amino acid deprivation might be similar, the deprivation of individual amino acids may induce different phenotypic and transcriptional responses; each individual amino acid participates in distinct metabolic pathways and different cells might have different intermediate demands. However, few studies have extensively profiled the global transcriptional response to a large number of amino acids. To that end, we systematically depleted each one of, or all of, the amino acids at a time to observe cellular phenotypic and transcriptional responses. We found that the deprivation of most amino acids, except glycine, triggered a robust and mostly conserved AAR. Quite unexpectedly, methionine deprivation triggered the most dramatic and extensive gene expression changes.

Methionine participates in multiple cellular metabolic pathways, including the salvage pathway, the SAM recycling pathway, the trans-sulfuration pathway for cysteine biosynthesis, polyamine synthesis, and creatine biosynthesis. Methionine, indirectly via SAM, also donates methyl groups for protein methylation, which can result in epigenetic changes when the proteins being methylated are histones. We dissected the contribution of each potential pathway to the cellular response of methionine deprivation and uncovered that creatine biosynthesis is essential for the methionine-deprived transcriptional response and histone modifications. Creatine biosynthesis depends on the availability of arginine and glycine; simultaneous deprivation of arginine or glycine abolished the methionine-deprivation transcriptional response and epigenetic changes. Together, our data reveal the heterogeneity of amino acid responses and a crosstalk among the metabolism of arginine, glycine and methionine in cells, in which arginine/glycine-dependent creatine biosynthesis is required for the methionine-deprivation response.

## Results

### Cellular and transcriptional responses to the deprivation of individual amino acids

To determine the cellular responses to different amino acids, we performed amino acid deprivation in the breast cancer cell line MCF7 that is positive for the estrogen receptor (ER) and has wild type p53 [12]. MCF7 cells were usually cultured and propagated in DMEM media that contained 15 amino acids, including 11 essential amino acids and 4 non-essential amino acids. To systematically define the global transcriptional response to the deprivation of any one (or all) amino acids, we prepared custom-made DMEM medias with the removal of one (or all) amino acids and supplemented with dialyzed fetal bovine serum. Similar approaches of depriving single amino acids have been previously applied by different groups to analyze the signaling pathways [13] and AARE-driven gene expression [14] of human cell lines.

**Fig 1:**
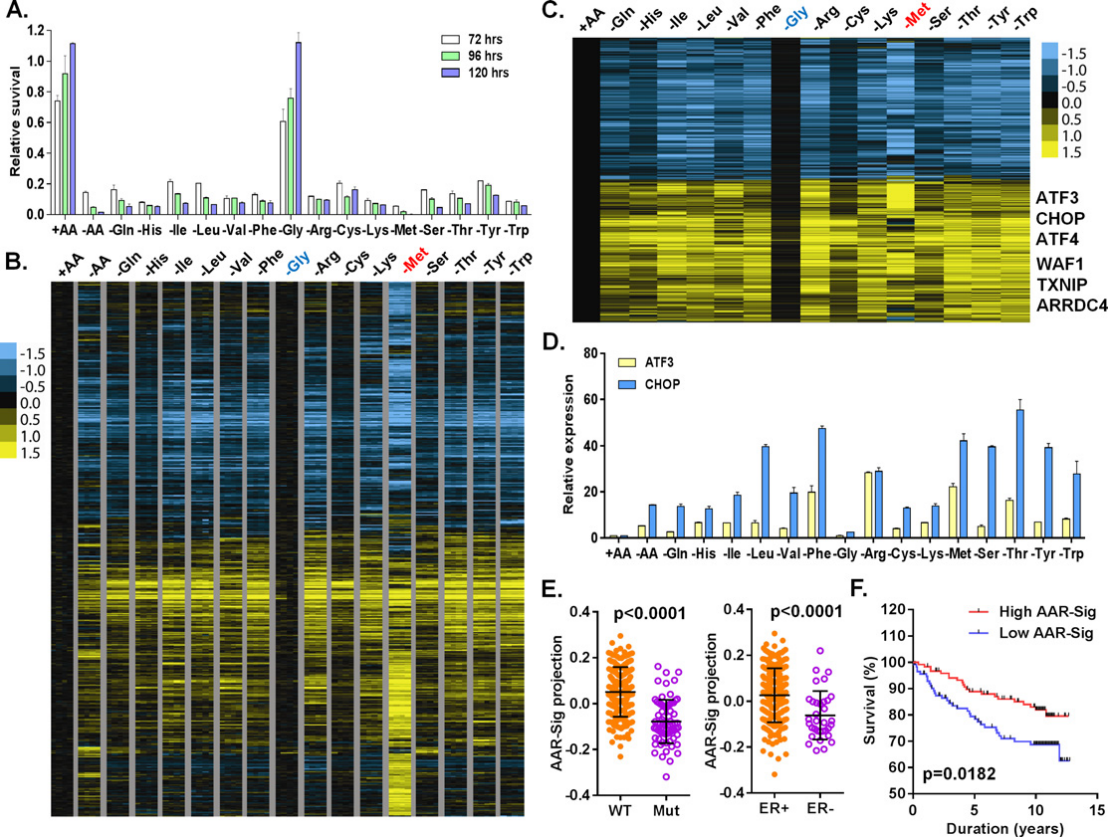
Cellular and transcriptional response to the deprivation of individual amino acids. (**A**) Relative cell survival of MCF7 cells upon the deprivation of all or indicated individual amino acids at the indicated times. (**B, C**) Heatmap of all (B) or common (C) transcriptional response of MCF7 in response to the deprivation of all or indicated individual amino acids. The samples of the deprivation of glycine (Gly, blue) and methionine (Met, red) were shown in the indicated colors. (**D**) The relative expression of ATF3 and CHOP in response to the deprivation of all or indicated individual amino acids. (**E**) The levels of the AAR signatures in the breast tumors when separated by wild type (WT) vs. mutant (Mut) p53 or positive vs. negative ER. (**F**) Kaplan-Meyer survival curves of Miller breast tumor sub-groups with either high or low associations with the common amino acid response gene signature.

First, we determined how the deprivation of each single amino acid affected the viability of MCF7 by crystal violet staining at different time points. During the experimental period of 72–120 hours, the viable cell number increased in the control media, while the deprivation of most individual amino acids dramatically reduced viable cell number (Fig 1A). The only exception to this was in glycine-free media where MCF7 cells proliferated to a similar degree as in control media, suggesting that MCF7 cells do not require extracellular glycine for growth. The deprivation of most amino acids reduced cell number to approximately the same extent, except for methionine deprivation, which led to the most dramatic reduction in cell number (Fig 1A). The deprivation of most amino acids led to moderate cell growth arrest within two days (S1A Fig). When compared to the deprivation of representative amino acids (leucine or glutamine), *propidium iodide* (PI) staining confirmed that methionine depletion caused the highest degree of cell death (S1B Fig).

Amino acid starvation generally triggers the transcriptional amino acid response (AAR) pathway and inhibits the nutrient sensing mTOR pathway. To examine the activities of these two pathways, we determined whether the phosphorylation of eIF2α (GCN2 activation in the AAR pathway) and S6K1 (T398, the mTOR sensing pathway) were affected by individual amino acid deprivation. Consistent with the lack of effects on proliferation, glycine deprivation did not affect pho-S6K1 (mTOR activity) or pho-eIF2α (GCN2 activity) (S1C Fig). In contrast, the deprivation of most other amino acids reduced phosphorylation of S6K (pho-S6K1) and increased phosphorylation of eIF2α (pho-eIF2a) to varying degrees. These results indicate that the deprivation of most individual amino acids, except glycine, triggered the expected canonical amino acid response.

To profile the transcriptional response to the deprivation of all or each of the 15 amino acids in DMEM, we exposed MCF7 to media with all (+AA), no amino acids (-AA) or missing one of each of the 15 amino acids, in quadruplicate, for 24 hours. The 68 RNA samples were then interrogated by Affymetrix U133A2 genechips to obtain global gene expression profiles (deposited into GEO with the accession number: GSE62673). First, we normalized all intensities of the 68 arrays by RMA, then mean-centered and filtered by 3-fold variation in at least two arrays to select 1365 probesets. These samples were then grouped based on their similarity of gene expression via unsupervised hierarchical clustering (S1D–S1F Fig). The control samples (+AA) clustered tightly with the glycine-deprived samples, consistent with the minimal effects on cell growth by glycine deprivation. This branch also contained His, Cys, Gln and Ser deprived samples (S1D–S1F Fig). The second branch contained three sub-branches: Val and Lys sub-branch; Ile, Leu and Phe sub-branch; and Arg, Thr, Tyr and Trp sub-branch (S1D–S1F Fig). The methionine (-Met) and all amino acid deprived samples (-AA) were in two distinct branches differentiated from all other amino acid deprived samples (S1D–S1F Fig). These data were in agreement with the clustered pattern analyzed by cross-correlation of individual AAR (S1G Fig). While not in perfect overlap, these clusters do have parallels with amino acid side chain characteristics: the nonpolar, branched amino acids (Val, Ile, Leu) were closely clustered.

We also found that many features existed in one or only a subset of amino acid deprived samples (S1F Fig). For example, deprivation of all, but not any one individual amino acid, induced the expression of calpain 9 (CAPN9), pre-B-cell leukemia transcriptional factor interacting protein 1 (PBXIP1) and C-C chemokine receptor type 4 (CCR4). Only serine deprivation induced the expression of RAB26 (a member of RAS oncogene family) and vesicle-associated membrane protein 1 (VAMP1), both of which are involved in vesicle trafficking. Arginine deprivation induced a specific set of genes that included chemokines (CCL1 and IL8) and kynureninase (KYNU), which catalyze the degradation of kynurenine. Kynurenine was previously found to be elevated in ER- breast tumors [15]. We also found many genes in the interferon responses induced in the samples deprived of methionine, arginine and lysine, with particularly high levels in the methionine samples (S1F Fig). Collectively, these results indicated that there is significant, previously-unappreciated heterogeneity in the transcriptional responses to the deprivation of different individual amino acids.

To define a net transcriptional response for each amino acid deprivation, we used a zero transformation process [16] to compare the changes in levels for each gene in each of 16 conditions (deprivation of one individual or all 15 amino acids) to the average transcript levels of the control treatment. 3741 probe sets were selected by the mean changes with at least ±2^0.8^ (~±1.74) fold change in more than three arrays and arranged using hierarchical clustering (Fig 1B). Such analysis revealed that the deprivation of most individual amino acids triggered a similar transcriptional response with the exception of glycine and methionine. Glycine deprivation triggered little transcriptional changes (Fig 1B), consistent with its lack of effects on proliferation (Fig 1A), signaling (S1C Fig) and co-clustering with the control samples in the unsupervised analysis (S1D–S1F Fig). In contrast, methionine deprivation triggered a distinct transcriptional response (Fig 1B) including extensive methionine-specific gene responses as well as aspects of the common amino acid deprivation response.

Next, we sought to define the common amino acid response (AAR) signature. Using the criteria of probe sets with at least a 2^0.8^ fold-change in more than three different individual amino acid deprivation samples, 778 probe sets were selected (S1 Table; Fig 1C). The common AAR gene signature contained many genes known to comprise the canonical amino acid response, including ATF4, ATF3, ASNS, SARS and CHOP (Fig 1C). We used real-time qPCR to validate the induction of ATF3 and CHOP mRNA when MCF7 was deprived of most individual amino acids, except glycine (Fig 1D). In addition to these canonic AAR genes, we also noted and validated the consistent induction of TXNIP and ARRDC4 in response to the deprivation of most individual amino acids (S1H Fig). Previous studies have suggested that glutamine deprivation and lactic acidosis affect glucose metabolism through the induction of TXNIP by the transcription complex MondoA/Mlx [17,18]. These results suggested that activation of TXNIP and ARDDC4 may also be part of the general amino acid response. The deprivation of most amino acids also induced p21 and MDM2, two well-established p53 target genes. Their induction was validated by qPCR and we found that these inductions were strongly reduced in p53-silenced cells (S1I Fig). Taken together, these data indicated that the deprivation of most individual amino acids activates the TXNIP and p53 pathways in addition to the canonical AAR.

To determine the *in vivo* relevance of the common amino acid response, we projected our derived common AAR gene signature (778 probe sets) to a breast tumor gene expression dataset with annotation of the p53 status [19]. We found that the common AAR gene signature was highly enriched in the ER and PR positive tumors (Fig 1E). The tumors with high enrichment of the common AAR gene signature were mostly associated with wild type p53 and low Elston grade (Fig 1E and S1J Fig). Furthermore, the tumors with high enrichment of the common AAR gene signature had better survival outcomes (Fig 1F). Together, these results support an anti-growth capacity of the common amino acid response and the role of TXNIP and p53 in tumor suppression.

### Unique and dramatic transcriptional response of methionine deprivation

**Fig 2:**
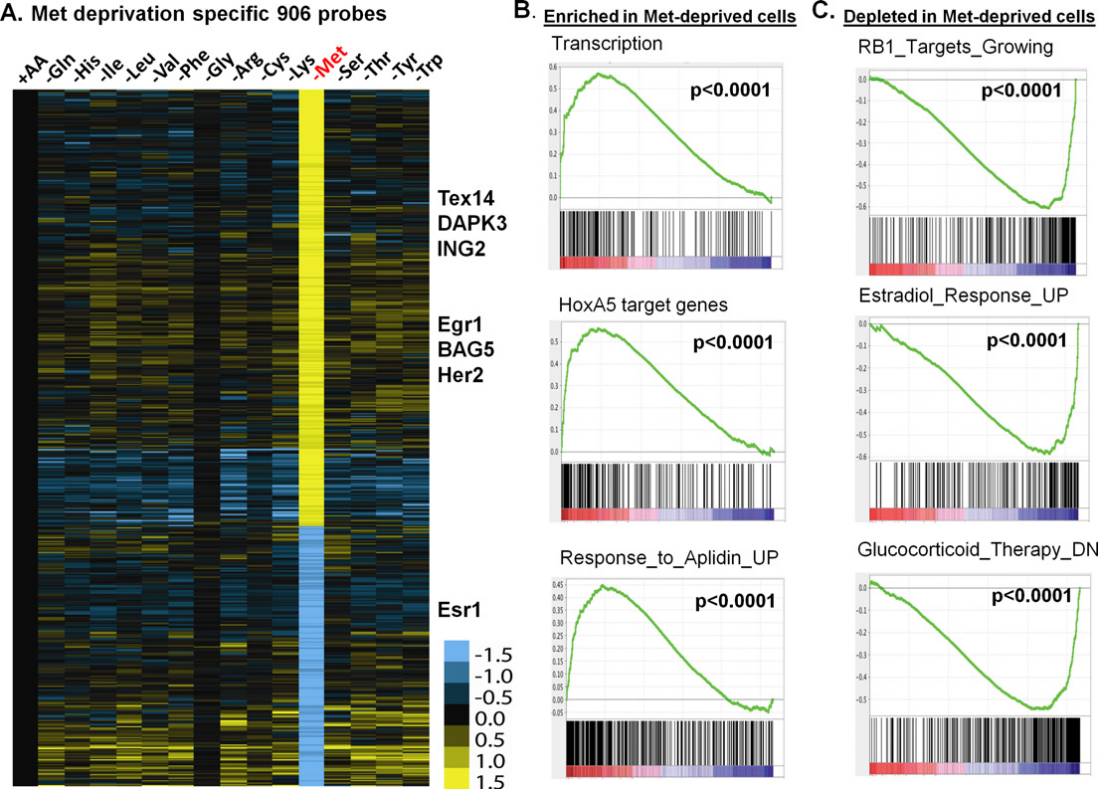
The methionine-deprived specific transcriptional response. (**A**) Heatmap of the 906 selected methionine-specific transcriptional changes in MCF7 cells in response to the deprivation of indicated all or individual amino acids. (**B, C**) GSEA analysis showed the enrichment (B) or depletion (C) of indicated genesets in the methionine-deprived cells.

Next, we identified individual amino acid-specific gene signatures based on the following criteria: at least ±2^0.8^ fold change of a probeset induced by the target amino acid deprivation relative to the mean transcript levels of the control samples, while less than ±2^0.5^ fold change of this probeset by all other amino acid deprivations (S2A Fig). Among all of the amino acids tested, methionine deprivation triggered the most dramatic and distinct transcriptional response (Fig 2A). Deprivation of most individual amino acids specifically affected very few probe sets (S2A Fig; S2 Table), such as 3 probe sets for glutamine deprivation and 39 probe sets for lysine deprivation. In contrast, we identified 906 specific methionine-specific probe sets that included 568 induced and 338 repressed probe sets (Fig 2A; S3 Table). When TRANSFAC was used to analyze the promoters of these induced methionine-specific genes, we noted an enrichment of the binding motifs of NRF2 (nuclear respiratory factor2), DEAF1 (Deformed Epidermal Autoregulatory Factor 1), GABP (GA binding protein) and E2F1 (S4 Table). In addition, 196 induced genes also have the predicted binding sites of activating transcription factor (ATF4) (S4 Table). This suggests that ATF4, that is triggered to mediate the canonical AAR (Fig 1), may also potentially contribute to the methionine-deprived specific response under methionine- deprived condition. The dramatic and distinct transcriptional response of methionine deprivation explained its distinct clustering pattern in the unsupervised analysis (S1D–S1F Fig).

To identify the biological pathways specifically affected by methionine deprivation, we performed Gene Set Enrichment Analysis (GSEA) by comparing methionine-deprived samples with samples deprived of several non-methionine amino acids, including arginine (Arg), isoleucine (Ile) and tyrosine (Tyr). When compared with arginine, the pathways enriched in the methionine-depleted samples included transcription (Reactome transcription, Pol III transcription initiation, KEGG-RNA-polymerase), telomere ends and maintenance, meiotic synapsis, HoxA5 target genes and genes in response to Aplidin (Fig 2B)[20]. In contrast, the pathways depleted in the methionine-depleted samples included RB1 targeted cell growth genes, progesterone and estradiol response genes, genes down-regulated by glucocorticoid therapy, oxidative phosphorylation and house-keeping genes (Fig 2C). Similar gene set or pathway enrichment or depletion patterns were seen when the methionine-deprived samples were compared with Ile or Tyr deprived samples. Additionally, we found that methionine deprivation suppressed ESR1, the gene encoding the estrogen receptor (S2B Fig). This observation was in agreement with depletion of estradiol response genes in the methionine-deprived samples (Fig 2C). The depletion of the house-keeping genes and highly expressed ESR1 and estrogen-dependent pathways in the ER positive MCF7 suggest that exogenous methionine is required to maintain the expression of these genes. In addition, we projected the methionine-deprived specific gene signature (MetDep-Sig, 906 probesets; S3 Table) to a CCLE gene expression dataset from 1037 cell lines with different tumor types [21]. Interestingly, we found that the MetDep-Sig was highly enriched in the cell lines originated from haematopoietic and lymphoid tissue (S2C Fig). The biological significance and the underlying mechanisms leading to such cell-type specific enrichment remain unclear. Together, methionine deprivation altered the expression of ER-dependent transcriptional program and other biological pathways in MCF7. In addition, methionine deprivation gene expression program was a prominent feature of haematopoietic malignancy.

### The transcriptional response of methionine deprivation is highly similar between MCF7 and PC3

To define the methionine concentrations at which methionine-specific responses were triggered, we exposed PC3 cells, a prostate cancer cell line, to different concentrations of methionine (from 10% to 0% of the regular DMEM that contains 200 μM methionine) for 24 or 48 hours. Using zero transformation analysis, we found that the methionine response could be triggered at 20 μM (10%) of methionine (S3A Fig), which are close to the levels found in human plasma [22]. Lower concentrations of methionine further enhanced the transcriptional response until the full methionine-deprivation response was triggered at 5μM (S3A Fig). When we examined a time course of the methionine-deprivation response between 24 or 48 hours, we did not note significant differences in the expression patterns (S3A Fig).

**Fig 3:**
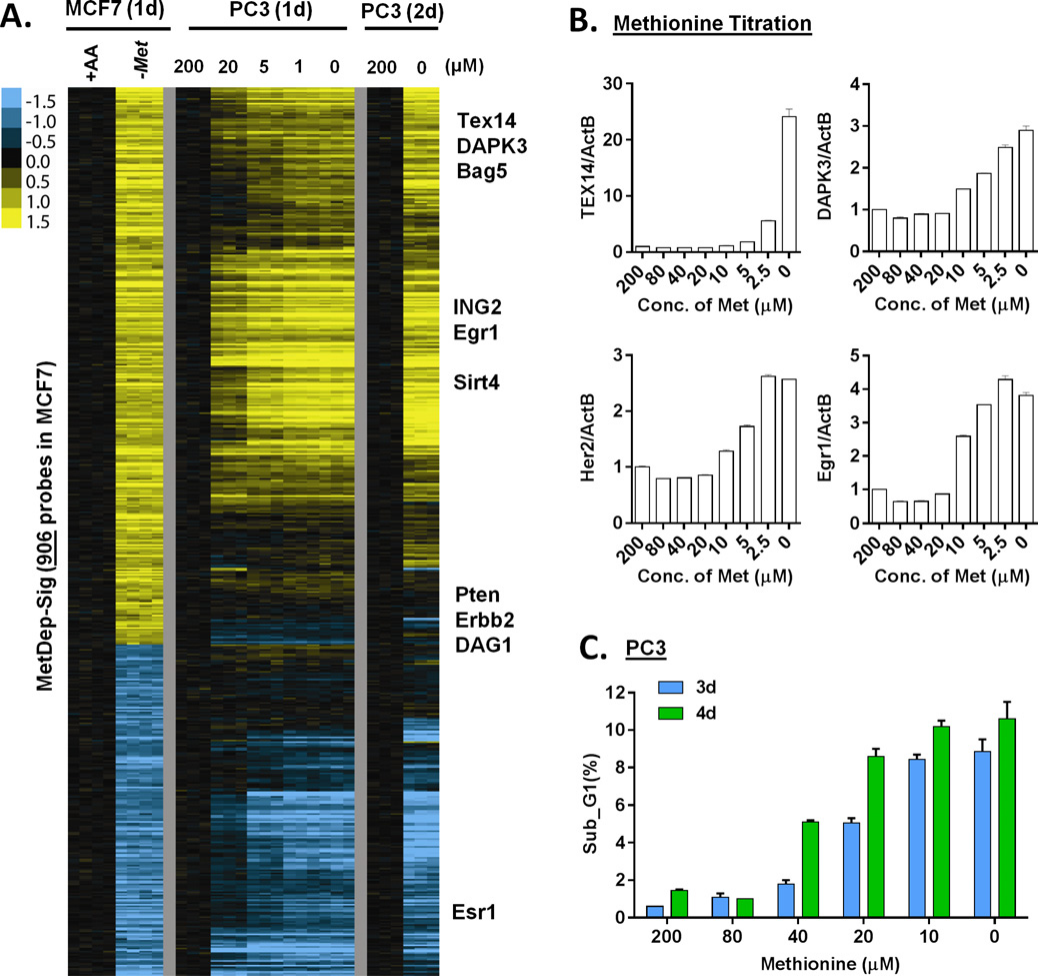
The transcriptional response of methionine deprivation between breast and prostate cancer cells. (**A**) Heatmap of the transcriptional changes of the genes in methionine-deprived specific gene response, defined in MCF7, in PC3 cells under different concentrations of methionine at indicated times. (**B**) Relative gene expression levels of TEX14, DAPK3, Her2 and Egr1 by qPCR in PC3 cells upon indicated different methionine concentrations for 24 hours (n = 3). (**C**) The percentage (%) of PC3 in the sub-G1 based on flow cytometry analysis of propidium iodide (PI) staining under indicated methionine concentrations for 3 or 4 days (n = 3).

To determine the cell-type specificity of the methionine-deprived responses, we compared the transcription response in MCF7 and PC3 cells. Since these two cell lines had different tissue origins, they had dramatically different basal gene expression profiles (S3B Fig). Remarkably, they had an overall highly similar transcriptional response to methionine deprivation (S3A Fig). Most of the 906 methionine-specific probe sets, defined in MCF7 cells, also showed concordant induction and repression in the PC3 cells (Fig 3A). We used real-time RT-PCR to validate the induction of a few methionine-deprivation induced genes, including TEX14 (Testis Expressed 14), DAPK3 (Death-Associated Protein Kinase 3), ING2 (Inhibitor of Growth Family, Member 2), BAG5 (Bcl-2-Associated Athanogene 5) and EGR1 (Early Growth Response 1). These genes were selected to represent the methionine-deprived specific response because their strong and reproducible induction under methionine deprivation. Also, these methionine-deprived genes encode proteins with a wide of variety of functions, including transcriptional regulation (EGR1 [23]), epigenetic regulation (ING2 [24]), spermatogenesis (TEX14 [25]) and apoptosis (DAPK3 [26], BAG5 [27]. While the functional implications of the induction of these genes by methionine deprivation remain unknown, we used these genes to dissect the regulation of methionine-specific gene response. In MCF7 cells, the reduction of methionine concentration to 10 μM (from 200 μM) started to trigger methionine-specific genes (Fig 3B), which was lower than the 20 μM observed for PC3 cells. These results suggested that the level of intracellular methionine needed to drop below a certain threshold to trigger the methionine transcriptional response, and that this threshold varies in different cell types. In the PC3 cells, more cell death was seen as methionine concentration was decreased (Fig 3C). Taken together, the methionine transcriptional response was triggered at 10–20 μM of exogenous methionine, and the induced transcriptional responses in MCF7 and PC3 were highly similar.

We also evaluated these gene responses in non-proliferating cells when grown in media containing 0.5% FBS. Under these conditions, we found that methionine deprivation still triggered the AAR and the methionine-specific gene response, but to a lesser degree (S3C Fig). In addition, when we examined how methionine deprivation (in 10%FBS) affects levels of the protein products of TEX14 and DAPK3, two methionine deprivation induced mRNAs, we did not observe the corresponding increase in protein levels (S3D Fig). These data suggested that methionine deprivation might also induce a profound inhibition of protein synthesis by the phosphorylation of eIF2α (S1C Fig). However, the lack of the protein induction did not affect our mechanistic studies of the transcriptional induction of these methionine-responsive genes.

### Methionine deprivation reduces histone methylation

**Fig 4:**
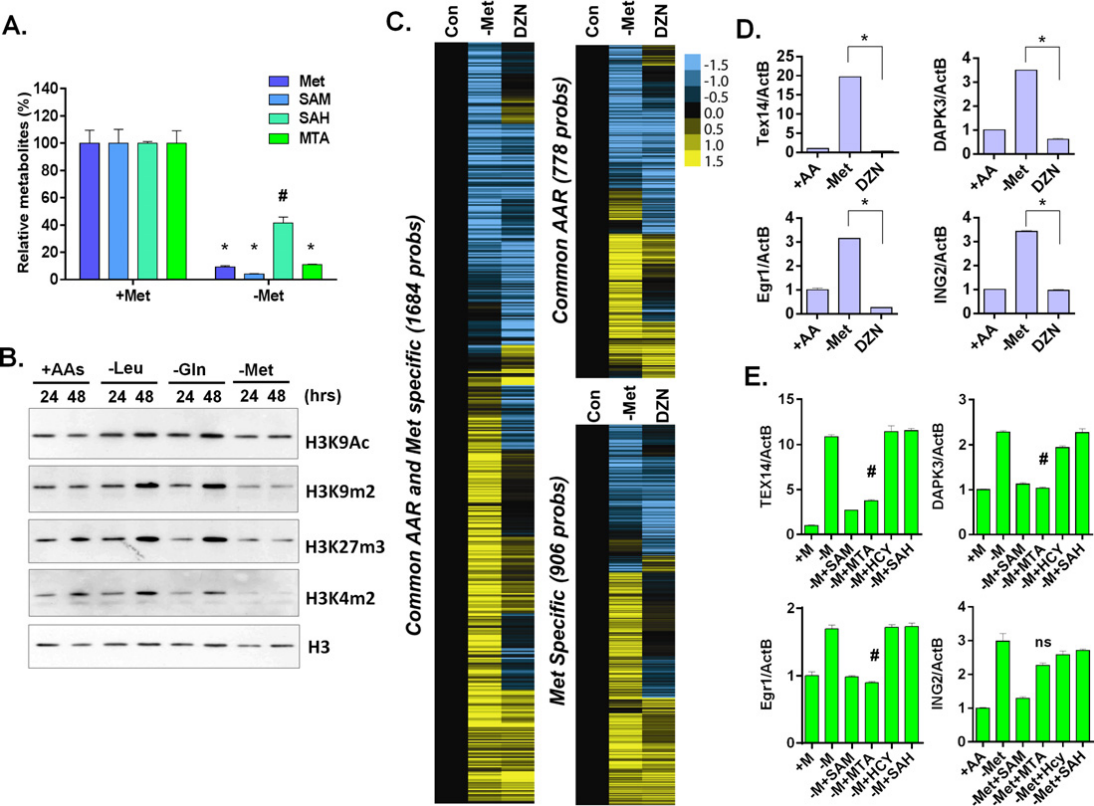
Methionine deprivation reduces the histone methylations. (**A**) Relative levels methionine, SAM, SAH and MTA in the MCF7 under control or methionine deprivation for 24 hours (n = 3; *, p < 0.001; #, p< 0.01). (**B**) Western blot analysis of indicated histone acetylation or methylations in MCF7 cells after 24 or 48 hours deprivation of Leu, Gln or Met. (**C**) Heatmap of the gene transcriptional changes in the methionine-specific and common amino acid response in MCF7 upon 5 μM DZNep (DZN) treatment. (**D**) Relative expression levels of TEX14, DAPK3, Egr1 and ING2 by qPCR in MCF7 cells after 24 hours of control (+AA), methionine deprivation (Met-) or 5 μM DZNep (DZN) (n = 3; *, p< 0.005). (**E**) Relative expression levels of TEX14, DAPK3, Egr1 and ING2 by qPCR in MCF7 cells after 24 hours methionine deprivation (-M) or supplemented with 200 μM of SAM, HCY, SAH or MTA respectively (n = 3; #, p< 0.05; ns, not significant).

Next, we sought to investigate how methionine metabolism affects the methionine-specific transcriptional response. The methionine recycling pathways consists of two branches: the *S-adenosyl-methionine* (SAM) cycle and the salvage cycle (S4A Fig). In the SAM cycle, methionine is recycled from SAM via S-adenosyl-homocysteine (SAH) and Homocysteine (HCY). In the salvage cycle, methionine is salvaged from 5'-methylthioadenosine (MTA) produced by polyamine biosynthesis that uses *decarboxylated* SAM (dcSAM) as substrates. In addition to methionine, methionine deprivation may affect the levels of other metabolites in the methionine recycling pathways. To evaluate this possibility, we used LC-MS/MS analysis and found methionine deprivation led to >90% depletion of SAM and MTA as well as a more modest depletion of SAH (Fig 4A).

S-adenosyl-methionine is a universal donor for the methylation reaction that modifies DNA, histones and other proteins. Therefore, it is possible that the methionine-specific gene expression may be due to changes in the epigenetic landscapes of the cells after methionine and SAM deprivation. Therefore, we used bisulfite pyrosequencing to determine whether a reduction of DNA methylation at the promoter regions of several methionine-deprived responsive genes might contribute to the induction of these genes. We tested seven genes, whose promoters are all located within CpG islands, and found no significant changes in DNA methylation at their promoter regions during methionine deprivation (S4B and S4C Fig). In addition, we examined the changes of global DNA methylation by LINE1 assay [28,29], and also found no significant changes on global DNA methylation during methionine deprivation (S4D Fig). Furthermore, to globally evaluate the potential contribution of DNA vs. histone methylation to the methionine-specific transcriptional response, we compared our methionine-deprived specific genes with the published datasets on the transcriptional response to the inhibitors of DNA methylation (5-AZA) and histone methylation (DZNep) in MCF7 cells (GSE17589)[30]. The methionine-deprived specific response overlapped more transcriptional response induced by the inhibitors of histone methylation (DZNep) than DNA methylation (S4E Fig). All these data suggested that alterations of DNA methylation did not play a major role for the methionine-deprived specific transcriptional response.

Consistent with the potential role of histone methylation, we noted that methionine deprivation reduced methylation modifications of several histone residues, including H3K4me2, H3K9me2, and H3K27me3 (Fig 4B). These results suggested a role for histone methylation in the transcriptional response to methionine deprivation. To determine this possibility in our own experimental system, we treated MCF7 cells with the EZH2 inhibitor 3-deazaneplanocin A (DZNep) that has been shown to be a global histone methylation inhibitor [31]. Microarray analysis indicated that DZNep treatment induced a robust gene response (S4F Fig) that had significant overlap with both the common AAR response and the methionine-specific transcriptional responses (Fig 4C and S4F Fig). For example, YY1, DICER1, and SOD2 genes were induced by both methionine deprivation and DZNep treatment (S4F Fig). However, there was also a portion of the methionine-deprivation induced signature, including Tex14, DAPK3, Egr1 and ING2, that was not induced by DZNep (Fig 4C and 4D). We also evaluated the levels of methylated histone in the promoter region of these genes during methionine deprivation. The level of H3K9me2 was high among the promoter regions of TEX14, DAPK3 and EGR1 genes and significantly dropped during methionine deprivation (S4G Fig, p<0.001). The level of H3K27me3 also slightly dropped on the promoter regions of TEX14 and EGR1 genes during methionine deprivation (S4G Fig, p<0.01). Therefore, loss of histone methylation is associated and may partially contribute to the transcriptional response of methionine deprivation.

To determine which branch(es) of the methionine recycling pathways might be responsible for the methionine-specific responses, we supplemented different metabolites (SAM, SAH or MTA) back to the methionine-depleted cells. SAM is a direct metabolic product of methionine and a substrate of both branches of the methionine recycling pathways. As expected, supplementation with SAM abolished the induction of all tested methionine-specific genes (Fig 4E). The supplementation of MTA (in the salvage cycle) also abolished most of the methionine deprived specific gene responses. In contrast, the supplement of SAH in the SAM cycle had no impact on gene induction (Fig 4E). Therefore, the depletion of SAM and MTA, and thus likely the salvage cycle, may play a more important role than SAH on the methionine-specific transcriptional responses in MCF7 cells. However, the result was tentative since we did not measure the cellular SAH level to determine the efficacy of SAH supplementation.

### Arginine is required for the specific gene response of methionine deprivation

**Fig 5:**
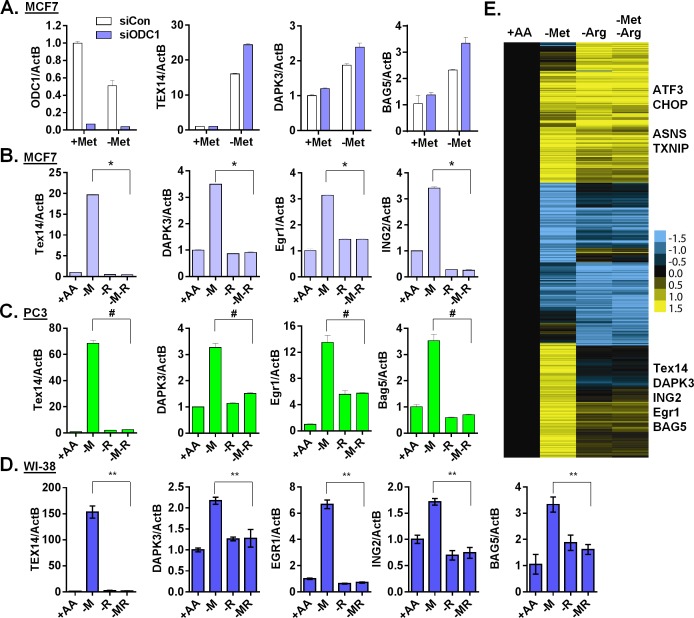
Arginine is required for the methionine-specific transcriptional response. (**A**) Relative expression levels of ODC1, TEX14, DAPK3 and BAG5 by qPCR in MCF7 transfected with siCon or siODC1 after methionine deprivation for 24 hours. (**B, C and D**) Relative expression levels of the indicated genes by qPCR in MCF7 (B), PC3 (C) or WI-38 primary fibroblasts (D) after depletion of either methionine (-M), or arginine (-R) or both methionine and arginine (-M-R) for 24 hours (n = 4; *, #, **, p < 0.01). (**E**) Heatmap of the global gene response in MCF7 cells after depletion of methionine (-M), arginine (-R) or both methionine and arginine (-M-R) for 24 hours.

In MCF7, the MTAP locus is deleted and the methionine salvage pathway is disrupted [32]. Therefore, the supplemented 5'-Methylthioadenosine (MTA) cannot be readily salvaged back to replenish methionine during methionine deprivation. Therefore, MTA could not abolish the methionine-specific response by simply restoring methionine levels. MTA is a byproduct of the polyamine biosynthesis that combines the decarboxylated S-adenosyl-methionine (dcSAM) (from SAM) and putrescine (from ornithine) to synthesize spermidine and spermine (S4A Fig). High levels of MTA inhibit polyamine biosynthesis [33]. Therefore, we reasoned that the supplementation of MTA might abolish the methionine-specific response by inhibiting the polyamine pathway. To test this possibility, we interrupted polyamine synthesis by inhibiting critical enzymes or removing its substrate arginine. We targeted the two key enzymes in the polyamine synthesis: ornithine cyclodeaminase (ODC1 that catalyzes the synthesis of putrescine from ornithine) and spermidine synthase (SRM that catalyzes the synthesis of spermidine from putrescine) to determine methionine-deprived specific responses. Surprisingly, we found that the inhibition of polyamine synthesis by genetically silencing ODC1 (Fig 5A) or SRM (S5A Fig) further enhanced the induction of TEX14, DAPK3 and BAG5 during methionine deprivation. Similarly, the ODC1 inhibitors POB and DFMO also did not abolish the methionine-deprived specific gene responses (S5B Fig).

Polyamine synthesis requires exogenous arginine to generate the immediate substrate, ornithine (S4A Fig). Therefore, we tested the relevance of exogenous arginine on methionine-specific gene response. Remarkably, simultaneous removal of both arginine and methionine almost completely abolished the methionine-specific gene response, including the induction of TEX14, DAPK3, EGR1 and ING2 in both MCF7 (Fig 5B) and PC3 cells (Fig 5C). Furthermore, this abolishment of the methionine-deprived specific gene responses was specific to arginine deprivation, since the simultaneous removal of glutamine or cystine (cysteine precursor) with methionine did not have similar effects (S5C Fig). Similar gene regulation patterns also occurred in the untransformed human primary fibroblast cells WI-38 and IMR90 (Fig 5D and S5D Fig). Interestingly, this dependence on exogenous arginine for the methionine-deprived response was consistent with our initial array analysis, in which all amino acids deprived samples also lacked the methionine-deprived response (S5E Fig). We then used microarrays to formally determine the effects of arginine deprivation on the global expression of MCF7 cells that were deprived of methionine, arginine or both methionine and arginine. We found that the induction of the 906 methionine-deprivation specific probesets was mostly abolished by the simultaneous removal of arginine (Fig 5E and S5F Fig). Together, our data indicated that exogenous arginine was required for the methionine-deprived specific gene response.

### Creatine biosynthesis is crucial for the methionine-deprived specific gene response

**Fig 6:**
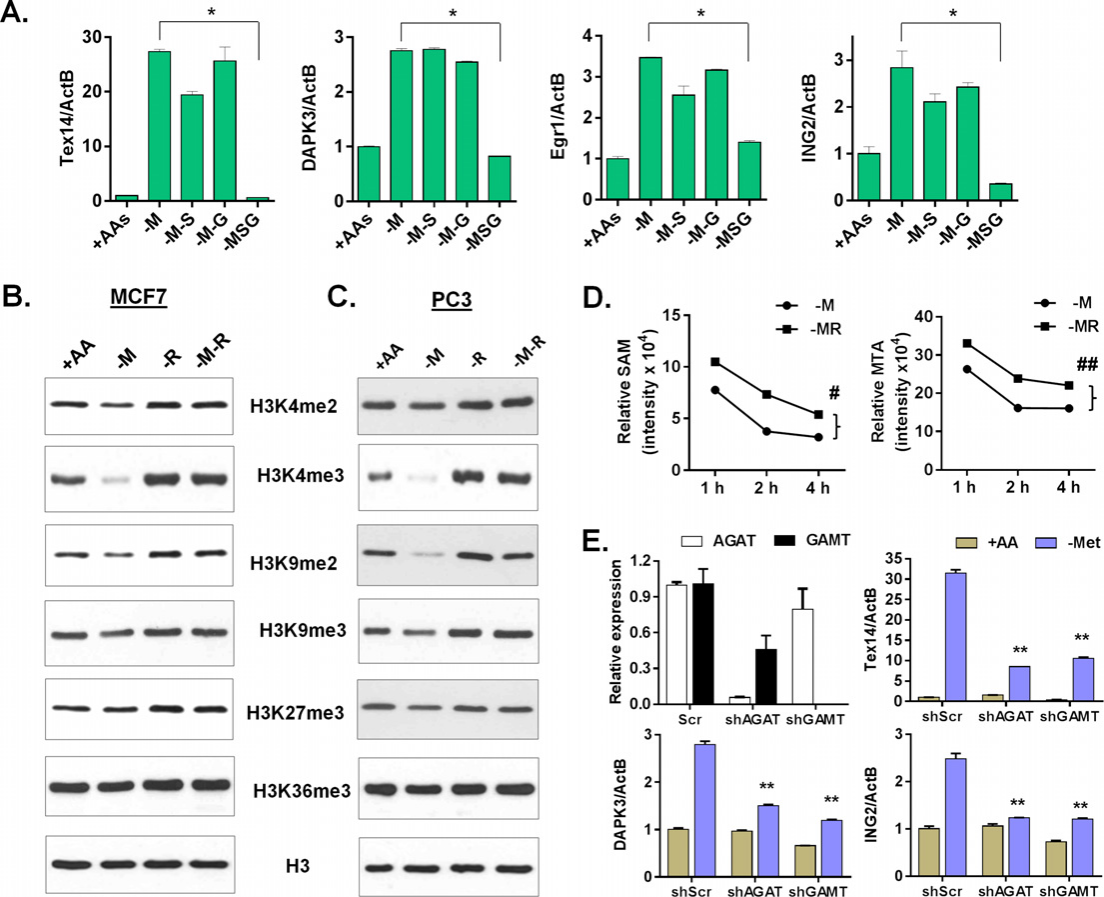
Arginine/glycine-dependent creatine biosynthesis is required for the reduction of histone methylation and the methionine-deprived specific gene response. (**A**) Relative expression levels of TEX14, DAPK3, Egr1 and ING2 by qPCR in MCF7 after depletion of either methionine (-M), or both methionine and serine (-M-S), or both methionine and glycine (-M-G), or methionine, serine and glycine (-MSG) for 24 hours (n = 3; *, p < 0.01). (**B, C**) Western blot analysis of histone H3 (control) and indicated histone methylations in MCF7 (B) or PC3 (C) after deprivation of methionine (-M), arginine (-R) or both methionine and arginine (-M-R) for 24 hours. (**D**) The levels of SAM and MTA in MCF7 cells after indicated time deprivation of methionine (-M), or both methionine and arginine (-MR) (n = 3; #, p < 0.01; ##, p< 0.01). (**E**) Relative expression levels of AGAT, GAMT, Tex14, DPAK3 and ING2 by qPCR in shRNA scramble (scr) and silenced AGAT (shAGAT) or GAMT (shGAMT) MCF7 cells after 24 hours of methionine deprivation (n = 3; **, p< 0.05).

Since the blockage of polyamine biosynthesis pathway was unable to abolish the methionine-deprived specific gene response, we investigated other metabolic pathways by which exogenous arginine may regulate the methionine-deprived gene response. In addition to polyamine synthesis, arginine also participates in the urea cycle, nitric oxide production and creatine biosynthesis (S4A Fig). We used inhibitors targeting metabolic enzymes in each of these arginine-dependent pathways. First, blocking the urea cycle by nor-NOHA (an arginase inhibitor) did not repress the inductions of methionine specific genes TEX14, DAPK3 and EGR1 (S6A Fig). Second, neither a NO scavenger (c-PTIO) nor inhibition of the nitric oxide synthesis (NOS) by L-NAME affected the methionine-specific gene responses (S6B Fig). These data mostly ruled out the role of the urea cycle and nitric oxide production as mechanisms by which arginine regulates the methionine-specific response. However, this conclusion was tentative since we did not validate the intended inhibition of arginase and NOS. Finally, we examined the role of the arginine-dependent creatine biosynthesis pathway. Creatine biosynthesis consists of two steps (S4A Fig): First, arginine and glycine are catalyzed by arginine:glycine aminotransferase (AGAT) to produce guanidinoacetate and ornithine. Second, guanidinoacetate *N*-methyltransferase (GAMT) transfers the methyl group from SAM to guanidinoacetate to yield creatine. Therefore, glycine is the other substrate required for the creatine biosynthesis. We considered whether the deprivation of glycine, similar to arginine, also affected creatine synthesis to abolish the methionine-specific response. However, glycine can readily be synthesized from serine by serine hydroxymethyltransferase (SHMT), consistent with the lack of cellular response to glycine deprivation (Fig 1). Therefore, we removed both serine and glycine to deplete intracellular glycine. As expected, the depletion of either serine or glycine alone had no impact on the induction of these genes during methionine deprivation (Fig 6A). However, depletion of serine and glycine completely abolished the induction of TEX14, DAPK3, EGR1 and ING2 upon methionine depletion in MCF7 cells (Fig 6A). In PC3 cells, co-depletion of serine and glycine with methionine was unable to abolish the methionine-deprived transcriptional response (S6C Fig), suggesting that there are alternative sources for the supply of glycine. In rodent and human, threonine can be synthesized to glycine [34,35]. Indeed, co-deprivation of threonine, serine and glycine completely abolished the induction of the methionine-deprived responsive genes (S6D Fig).

The use of SAM as a substrate for creatine biosynthesis has been considered a major SAM-consuming reaction [36,37]. Therefore, interrupting creatine biosynthesis by removing either arginine or glycine possibly slows down the SAM depletion and the resulting histone demethylation. Indeed, we found that the levels of several histone methylations, such as H3K4me2, H3K4me3, H3K9me2, and H3K9me3 decreased during methionine deprivation, but were restored by the co-deprivation of methionine and arginine in both MCF7 and PC3 cells (Fig 6B and 6C). In contrast, the reduction of H3K27me3 and H3K36me3 under methionine deprivation was modest, and sometimes inconsistent, in both MCF7 and PC3 cells. The reduction of H3K4me3 during methionine deprivation was also fully restored by the simultaneous deprivation of both serine and glycine in MCF7 and of all three threonine and serine and glycine in PC3 cells (S6E Fig). We also evaluated the levels of related intracellular metabolites during deprivation of methionine, arginine, and both methionine and arginine. Consistently, methionine deprivation reduced the levels of intracellular methionine, SAM, SAH and MTA (S6G Fig). Co-depletion of arginine and methionine maintained significantly higher levels of SAM and MTA than with methionine deprivation alone (Fig 6D, S6F and S6G Fig; p < 0.01). In addition, arginine depletion significantly reduced intracellular ornithine (p < 0.001), but not creatine (S6G Fig). Genetic silencing of either AGAT or GAMT by shRNA to interrupt the creatine biosynthesis also significantly repressed the induction of methionine deprived specific genes (Fig 6E and S6H Fig). Therefore, our data suggested that arginine- and glycine-dependent creatine biosynthesis consumes intracellular SAM to reduce histone methylation and cause a methionine-deprived specific gene response.

### Creatine biosynthesis contributes to epigenetic changes and ornithine-mediated signaling in the methionine-deprived specific gene response

**Fig 7:**
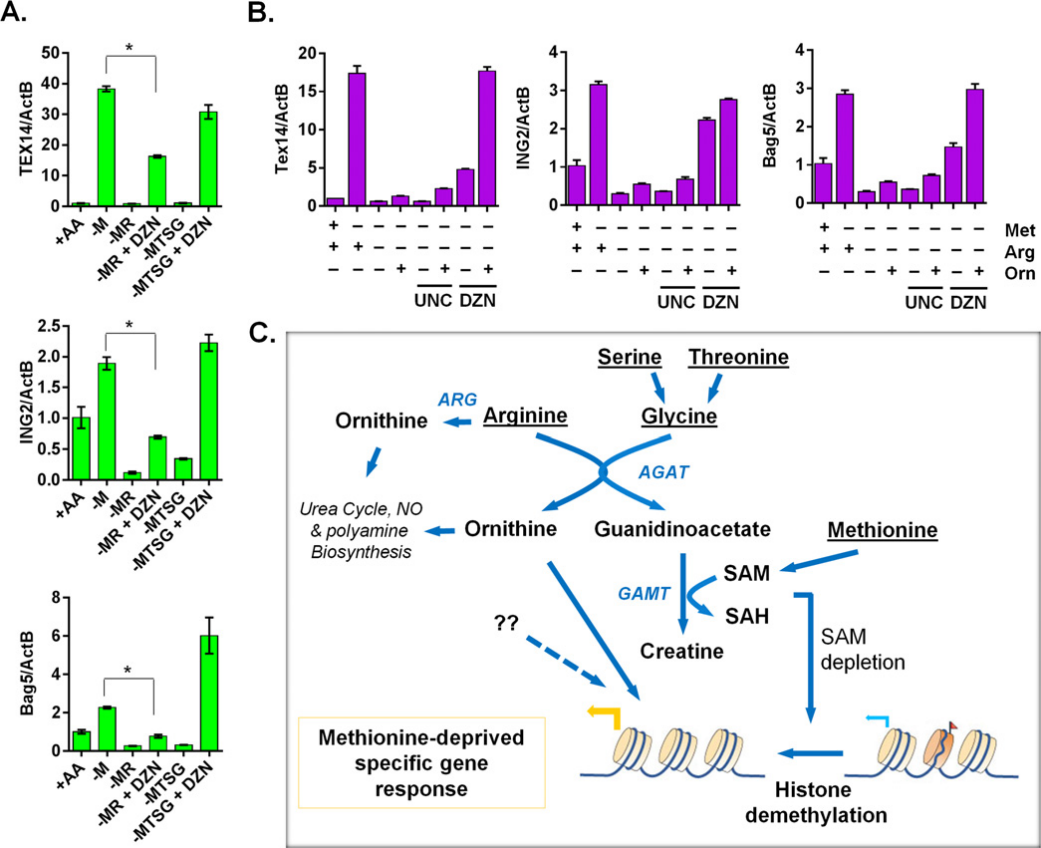
The ornithine-mediated signaling is required for the complete induction of the methionine-deprived specific gene response. (**A**) Relative expression levels of TEX14, ING2 and Bag5 by qPCR (n = 3; *, p < 0.01) in PC3 cells after 24 hours depletion of either methionine (-M), methionine and arginine (-MR), or methionine, threonine, serine and glycine (-MTSG) with or without DZNep (DZN, 5 μM). (**B**) Relative expression levels of Tex14 and ING2 by qPCR in MCF7 cells after 24 hours depletion of methionine (-M), methionine and arginine (-MR) under different combination with ornithine (Orn, 400 μM), UNC0638 (UNC, 10 μM), DZNep (DZN, 5 μM). (**C**) The model of the crosstalk between arginine/glycine-dependent creatine biosynthesis and methionine-deprived specific transcriptional response.

Since intact creatine biosynthesis was required for reduction of histone methylations during methionine deprivation, we examined whether the inhibitors of histone methylation was able to restore the methionine-deprived specific gene response in the context of blocked creatine biosynthesis. Indeed, the general histone methylation inhibitor DZNep (DZN) fully restored the induction of TEX14, ING2 and BAG5 genes when creatine biosynthesis was prevented by co-depleting serine, threonine and glycine (the glycine branch of creatine synthesis) (Fig 7A). However, DZN only partially restored the induction of the methionine-deprived specific genes when the arginine branch of creatine biosynthesis was blocked (Fig 7A). Besides being used for creatine biosynthesis, arginine can be synthesized to ornithine, as arginine deprivation reduced the level of intracellular ornithine (S6G Fig). We hypothesized that ornithine-mediated signaling was also required for the full induction of methionine-deprive specific gene response. Indeed, addition of ornithine with DZNep rescued most of the methionine-deprived specific gene response when the creatine biosynthesis was abrogated by arginine depletion (Fig 7B). We also examined UNC0638 (UNC), a specific inhibitor of methyltransferases G9a and GLP, which are responsible for H3K9 di-methylation. We found that UNC had no significant rescuing effects on the induction of methionine-deprived specific genes (Fig 7B), suggesting that demethylation of H3K9me2 alone may be not sufficient for the observed methionine-deprived transcriptional changes. The addition of ornithine enhanced the induction of some genes in the context of methionine deprivation alone, but ornithine alone was unable to rescue the induction of the methionine-deprived specific genes when the creatine biosynthesis was blocked by arginine depletion (S7A Fig). Creatine had similar enhancement effects as ornithine on the methionine-deprived gene response (S7B Fig). Taken together, our data suggested a model in which an intact arginine- and glycine-dependent creatine biosynthesis contributes to SAM depletion and resulting epigenetic changes. The combination of these epigenetic alterations and ornithine-mediated signaling are required to fully induce a specific methionine-deprived transcriptional response (Fig 7C). Based on these data, we propose a model that the intact arginine- and glycine-dependent creatine biosynthesis contributes to two events in the methionine-deprived specific gene response: depletion of SAM for epigenetic changes and maintaining ornithine-mediated signaling (Fig 7C).

## Discussion

In this study, we have comprehensively profiled the transcriptional responses to the deprivation of 15 individual amino acids. While the deprivation of most amino acids triggered the canonical amino acid response (AAR), we further identified unexpected features and heterogeneities of the amino acid deprivation responses. Based on our observations, we propose a model of a shared amino acid response (AAR), as well as a distinct methionine-specific response. The shared AAR was mediated by at least three different pathways, including the well-defined canonical AAR, p53 and TXNIP pathways. The unique and extensive transcriptional response to methionine deprivation was dependent on both epigenetic changes and the ornithine-mediated signaling events. We showed that methionine deprivation depleted cellular SAM and histone methylation. Importantly, we determined that arginine- and glycine-dependent creatine biosynthesis was required for the methionine-deprived specific response due to its role in SAM consumption. Collectively, our data revealed previously unappreciated heterogeneity among individual AARs and a distinct methionine-deprivation gene response that resulted from the crosstalk between arginine, glycine and methionine metabolism through creatine biosynthesis and ornithine-mediated signaling.

In addition to the induction of a canonical AAR, we found that the deprivation of most amino acids also leads to a consistent activation of p53 and induction of TXNIP. Therefore, the activation of p53 and TXNIP should be considered additional branches of the common AAR, at least in cells with intact p53. Activation of these programs by amino acid deprivation was reported by isolated previous studies. For example, serine deprivation can activate p53 and induce metabolic reprogramming [38]. Here, our results showed that p53 activation seems to be a general feature in the response to the deprivation of most amino acids and therefore suggests that the p53 status of tumor cells may affect their response to amino acid deprivation. One potential mechanism by which an AAR could activate p53 is through the ribosomal stress response [39]. Due to an excess of uncharged tRNAs, an imbalance of the ribosomal biogenesis results and the released ribosomal proteins bind and inhibit MDM2 to activate p53 and halt cell cycle progression [39]. Therefore, p53 activation, together with mTOR inhibition, may both contribute to the arrest of cellular proliferation as energy conservation mechanisms during amino acid deprivation.

Under glutamine deprivation, the induction of MondoA-TXNIP activates glucose uptake to compensate for the limited availability of another carbon source [17]. The MondoA-Mlx transcription complex plays a pivotal role in glucose homoeostasis by activating TXNIP and limiting glycolysis in response to glucose 6-phosphate, adenine nucleotides and acidosis [18,40]. An ATF5-TXNIP axis was also suggested to switch from an adaptive UPR from ER stress to a terminal UPR and cell death [41]. It remains to be determined whether MondoA or ATF5 play a major role in the TXNIP induction associated with amino acid deprivation. Given the well-recognized role of TXNIP to repress glucose uptake and glycolysis [42], we expect that amino acid deprivation may also affect glucose metabolism in cancer.

Among different amino acids, our most striking result was that methionine deprivation induced a unique transcriptional response. Since SAM, a product of methionine catabolism, is a major donor of methyl groups for various epigenetic modifications in cells, we hypothesize that its depletion would result in epigenetic changes, and that these changes would play a role in the methionine-specific transcriptional responses. Through investigation of this hypothesis we discovered that histone methylation, rather than DNA methylation, played a significant role in the methionine-specific response. We noted a marked reduction in the levels of histone methylation, but very little difference in the levels of DNA methylation on relevant gene promoters and global DNA methylation. In addition, global inhibition of histone methylation, but not DNA methylation, induced a transcriptional response that had significant overlap with the methionine-deprived gene response. While this may reflect the different turnover mechanisms and dynamics of DNA and histone methylation, it also suggested an important role for histone methylation in the cellular response to methionine deprivation. Since the epigenetic landscape of a cell, defined in part by the DNA and histone methylation, contributes greatly to tissue-specific expression patterns, however, we observed that the methionine-deprived gene response did not exhibit much tissue-specific features. Our results of reduced histone methylation are consistent with a recent study that shows methionine deprivation of human ES cells reduced tri-methylation of histone H3 lysine-4 (H3K4me3), an active mark that is crucial for maintaining the stem cell fate [43]. In the mouse ES cells that depend on the threonine for SAM levels, threonine deprivation also reduced the level of H3K4m3 [35]. Very intriguing, H3K4me2,3, as informative as activating methylation marks generally associated with active gene transcription[44], are largely reduced during methionine deprivation. We speculate that induction of the methionine-deprived specific transcription response may be due to rearrangements of reduced both active and repressive histone marks, not solely relying on either one. Additionally, we observed a highly similar methionine-deprivation transcriptional response in MCF7 (breast) and PC3 (prostate) cells, regardless of their different tissues of origin and dramatically different basal levels of gene expression. These data suggested that shared signaling events, in addition to epigenetic alterations, play an important role in the methionine-specific responses. We determined that at least one “second” signal is mediated by ornithine.

Methionine and its metabolic derivatives participate in several diverse metabolic pathways, including the biosynthesis of polyamines, glutathione, purines, and creatine. We identified that creatine biosynthesis was particularly crucial for the methionine-deprived specific gene response. Since both arginine and glycine are substrates for the creatine biosynthesis, the deprivation of either amino acid blocks creatine biosynthesis and abolishes the methionine-deprivation transcriptional response. We determined that the large consumption of SAM during creatine biosynthesis contributed significantly to the SAM depletion during methionine deprivation. Indeed, the co-deprivation of arginine and glycine, to block creatine biosynthesis and delay SAM depletion, allowed for the maintenance of histone methylation under methionine deprivation. In addition, we found that ornithine-mediated signaling was also required for the complete induction of the methionine-deprived transcriptional response. This signaling may account for the similar methionine-deprived transcription response that occurred in cells with different origins. We propose a model in which the intact arginine- and glycine-dependent creatine biosynthesis contributes to two events for the methionine-deprivation specific gene response: depletion of SAM for epigenetic changes and maintaining ornithine-mediated signaling (Fig 7C). There may be other factors that contribute to the methionine-deprived specific transcriptional responses, such as ATF4 or other transcriptional factors. This model reveals the importance of the crosstalk between arginine, glycine and methionine metabolism for methionine-deprivation mediated biological consequences. These data are also highly relevant for developing chemical strategies to target both pathways to fully replicate the benefits of methionine deprivation.

Our studies do have limitations. First, we focused on early time points within the first 1–2 days of methionine deprivation. At this time point, the main epigenetic changes occur at the histone methylation levels (Figs 6B and S4D). It is possible that long-term methionine deprivation may lead to additional changes, such as DNA methylation. Second, it will be important to consider the range of methionine levels in plasma and tissues to determine the relevant range of responses under varying methionine levels during physiological and pathological adaptations. Importantly, we did observe significant gene expression changes triggered around the physiological levels of methionine in plasma (~20 μM) [22]. Third, while we used several specific methionine-deprived genes to represent methionine-deprived specific responses to study their regulatory mechanisms, the results may be confined to these genes unless we have followed up with global array studies, such as the co-depletion of methionine and arginine (Fig 5E).

Several studies of methionine restriction in human and rodents have found that methionine deprivation can lower the plasma methionine levels and slow tumor growth in rodent models [45–47]. Therefore, methionine restriction may be an important strategy to treat the cancers that exhibit a dependence on methionine for survival and proliferation. We found that methionine deprivation activates the transcription of HoxA5 target genes. Compromised HOXA5 function can limit p53 expression in human breast tumors and overexpression of HOXA5 can induce tumor cell death and decrease invasive abilities of lung tumor cells [48]. Additionally, the methionine-deprivation signature was enriched for genes activated in response to Aplidin, a marine organism-derived compound with potent anti-myeloma effects that is currently in clinic trials [20]. The enrichment of HOXA5- and Aplidin-induced genes in the methionine-deprived samples may be consistent with the anti-tumor potential of methionine deprivation. Methionine dependence in cancer may also be due to one or a combination of deletions, polymorphisms or alterations of genes involved in the *de novo* and *salvage* pathways. Cancer cells with these defects in methionine metabolism are unable to regenerate methionine, and thus are addicted to exogenous methionine for survival or proliferation.

Mostly interestingly, methionine restriction can increase longevity across species, including yeast, flies and rodents [49–51]. In addition to general mTOR inhibition by amino acid restriction, methionine restriction likely has unique features to extend lifespan, which are under active investigations [52,53]. Our data reveal that methionine restriction causes a unique methionine-deprived transcriptional response in the context of intact arginine/glycine dependent creatine biosynthesis. Supplements of arginine, or its derivatives ornithine and creatine, all of which are metabolites in the creatine biosynthesis pathway, have beneficial anti-aging effects, such as reduced risk of vascular and heart disease, reduced rate of erectile dysfunction, improvement in immune response and inhibition of gastric hyperacidity [54–56]. Our data indicates that methionine deprivation induced its unique gene response through a reduction of both histone methylation and ornithine-mediated signaling. It remains to be seen if the anti-aging effect of methionine restriction in vivo is associated with histone demethylation and/or ornithine-mediated signaling. It would be interesting to test in organisms whether additional arginine restriction abolishes the beneficial effects of methionine restriction on lifespan extension and examine whether the defined methionine-deprived gene signature is enriched in methionine-restricted animal tissues or in long-lived human individuals.

Based on our current understanding of the consequences of methionine restriction, manipulating the level of methionine in our bodies may have beneficial roles for cancer control and anti-aging. Since the main source of methionine for humans is from food, one strategy to lower in vivo methionine levels would be to restrict or remove methionine from the diet. Vegan diets, which have lower levels of methionine, may therefore be a useful nutritional strategy to combat cancer growth and extend lifespans. Alternatively, plasma methionine levels could be reduced in vivo by methioninase, which degrades methionine [57], similar to using asparaginase to deplete plasma asparagine levels in the treatment of ALL. Our studies present the mechanisms underlying a unique methionine-deprived transcriptional response, which may provide useful insights to understand the nature of methionine restriction for cancer control and lifespan extension.

## Materials and Methods

### Cell culture and individual amino acid deprivation

MCF7 breast cancer cells and PC3 prostate cancer cells were cultured in Dulbecco's modified Eagle's medium (DMEM; GIBCO-11995) supplemented with 10% fetal bovine serum and 1 × antibiotics (penicillin, 10,000 UI/ml and streptomycin, 10,000 UI/ml). To prepare amino acid deficient media, Earle’s balanced salt solution was added with 4.5 g/L glucose (Sigma-Aldrich), MEM vitamin solution (Invitrogen), 0.37mM sodium bicarbonate (Sigma-Aldrich), 24.8 μM ferric nitrates (Sigma-Aldrich), with deficiency of one (or all) amino acid, and supplemented with 10% dialyzed FBS (10,000 MW cutoff, Sigma (F0392)) and 1X antibiotics. The cells were maintained in a humidified incubator at 37°C and 5% CO_2_. Additional materials and methods were listed in S1 Text.

### Cell proliferation and death assays

Relative cell number was monitored by crystal violet staining. Triplicate samples of 10^4^ MCF7 cells were seeded in 96 well plates. After 24 hours, the medium was removed and cells were washed twice with 1X PBS. The cells were then treated with individual amino acid deprivation media or the control full media. After the appropriate time period, the cells were fixed and stained with 1% crystal violet solution. With extensive washing, crystal violet was resolubilized in 10% acetic acid and quantified at 595 nm as a relative measure of cell number. Alternatively for propidium iodide staining method, the cells treated with the indicated amino acid deprivation were trypsinized and collected, fixed in ice cold 70% ethanol overnight. Cells were washed twice with 1XPBS buffer and resuspended in PBS buffer containing 50 μg/mL propidium iodide (PI) and 10 μg/ml RNase A. Flow cytometry was carried out using BD FACSCanto II flow cytometer. At least 10,000 cells were analyzed per sample.

### RNA isolation and microarray analysis

RNA from MCF7 cells exposed to the control, one (or all) amino acid deprivation conditions (four replicates in each condition) for 24 hours or RNA from PC3 cells exposed to different concentration of methionine (three replicates in each condition) for 24 hours or 48 hours were extracted by RNAeasy kits (Qiagen) and hybridized to Affymetrix human genome 133A 2.0 arrays with standard protocol. Similar methods were applied on PC3 RNA analysis. The data was deposited in NCBI GEO site (GSE62673).

### Data analyses

Affymetrix Probe intensities were normalized as log_2_ value by RMA and the expression data were subjected to unsupervised hierarchical average linkage clustering using Cluster 3.0 and displayed using TreeView. For supervised clustering analysis, the changes of gene expression upon one (or all) amino acid deprivation were derived by zero-transformation (Δlog_2_) against the control condition. The probesets that varied from the baseline by 2^0.8^ fold in at least 4 chips were selected for hierarchical clustering. To observe common amino acid deprivation gene response, 778 probes were selected by 2^0.8^ fold varied from the control samples in at least 4 different individual amino acid deprivations. To identify the methionine deprived specific gene response, 906 probes were selected in methionine deprivation samples by 2^0.8^ fold varied from the control samples, while no more than 2^0.5^ fold changes in other individual amino acid deprivation.

The common AAR gene signature “R-value” projection analysis was performed according to a previous report [58]. For example, the common AAR gene signature (using ‘‘1” and ‘‘-1”, for up and down, respectively) was listed in S1 Table. To score each tumor within a dataset for similarity to common AAR gene signature, we derived an R-value for each tumor in correlation to common AAR gene signature. The R-value was computed as the Pearson’s correlation between the pattern of common gene signature and the tumor’s expression dataset that were firstly normalized by the mean. In this way, tumors with high R-values would tend to have high similarity with common AAR gene signature. Data were analyzed using *Gene Set Enrichment Analysis (GSEA)* as described using indicated selection criteria.

### Real-time RT-PCR and DNA methylation and chromatin immunoprecipitation (Chip) qPCR assay analysis

RNA was reverse-transcribed to cDNA with the SuperScript II reverse transcription kit using random hexamers. The gene expression level was measured by quantitative PCR (qPCR) with Power SYBR Green PCR Mix (Applied Biosystems) following the manufacturer's protocol.

Genomic DNA was extracted from MCF7 cells using the QiaAmp DNA mini-prep kit according to the protocol provided by the manufacturer (Qiagen). The genomic DNAs (800 ng) were modified by treatment with sodium bisulfite using the Zymo EZ DNA Methylation kit (Zymo Research, Irvine, CA). Bisulfite treatment of denatured DNA converts all unmethylated cytosines to uracils, leaving methylated cytosines unchanged, allowing for quantitative measurement of cytosine methylation status. Pyrosequencing was performed using a Pyromark Q96 MD pyrosequencer (Qiagen). The bisulfite pyrosequencing assays were used to quantitatively measure the level of methylation at CpG sites contained. Assays were designed to query CpG islands using the Pyromark Assay Design Software (Qiagen). PCR was performed using the following conditions: 95ns: 95following conditions: 95ions: 95 metet level of metl of metl of meteasure the level of mete level of metel of metevel of mett of cytosPyrosequencing was performed using the sequencing primer. PCR conditions were optimized to produce a single, robust amplification product. Defined mixtures of fully methylated and unmethylated control DNAs were used to show a linear increase in detection of methylation values as the level of input DNA methylation increased (Pearson r > 0.98 for all regions). Once optimal conditions were defined, each assay was analyzed using the same amount of input DNA from each specimen (40 ng, assuming complete recovery after bisulfite modification). Percent methylation for each CpG cytosine was determined using Pyro Q-CpG Software (Qiagen).

Chromatin immunoprecipitations were performed using digested chromatin from MCF7 cells according to the protocol of SimpleChIP Enzymatic Chromatin IP Kit (Cell signaling, #9002). Purified DNA was analyzed by qPCR methods. All primers used in this study were listed in S5 Table.

### Metabolomics reagents and methods

This section contains detailed materials and methods for metabolomics data shown in Fig 4A (high resolution LC-MS), Figs 6D, S6F and S6G (Quantitative LC-MS/MS).

#### Reagents

The following stable-isotope internal standards were used for this study: amino acid standard mix NSK-A containing methionine, arginine, ornithine, isoleucine, and phenylalanine (Cambridge Isotope Laboratories), d3-creatine (Sigma Aldrich), and d3-5’-methylthioadenosine (13C Molecular, Fayetteville, NC).

#### Sample preparation for metabolomics

To each cell pellet, 100 μl of 50 mM Ammonium Bicarbonate pH 8 (AmBic) was added. Cells were ruptured by probe sonication with three five-second continuous bursts, with cooling on ice between cycles. A protein assay (mini-bradford, Bio-Rad, Inc) was used to approximate total biomass, diluting 10 μL of the crude lysate by 10x in AmBic. For the high resolution (Fig 4A) time course quantification LC-MS/MS (Figs 6D and S6F) studies, crude lysates were then concentration-normalized in a 96-well plate to contain approximately 600 μg protein using the Bradford assay data, and then diluted 5x with MeOH containing 2.5 μM of NSK-A as internal standards. For the 3-hour quantitative study (S6G Fig), crude lysates were normalized to approximately 1mg protein in 100 uL in a well-plate, to which 900 ul of MeOH containing 1.67 uM NSK-A, 1.67 uM d3-creatine, and 0.538 nM d3-MTA was added. The MeOH:AmBic extracts were incubated on a Thermomixer R (Eppendorf) for 30 minutes at 32°C with gentle mixing. The metabolite extract was then centrifuged at 3000 rpm to pellet protein and other solids, and the MeOH-soluble extract was transferred to clean wells and dried under N2. Samples were resuspended in 100 μl containing 89/10/1 v/v/v water/MeCN/TFA, mixed and centrifuged briefly, samples were analyzed directly from the well plate.

#### Metabolite profiling by liquid-chromatography mass spectrometry

Relative quantitation of arginine (Arg), methionine (Met), 5’-methyl thioadenosine (MTA), s-adenosyl methionine (SAM), s-adenosyl homocysteine (SAH), between normal (+Met) and methionine-depleted (-Met) conditions was first performed using a chromatographic method specifically developed for these compounds, in the context of an unbiased high-resolution LC-MS/MS analysis under the two conditions. Two microliters of sample was injected onto a 150 μm x 10 cm iKey (Waters Corporation) with 1.7 μm BEH C18 particles. Mobile phase A was 0.01/0.1/99.89 v/v/v HFBA/formic acid/water, and mobile phase B was 0.1% formic acid in acetonitrile. The analytical gradient was performed at a flow rate of 2 μl/min and 45°C column temperature, as follows: 2 minute hold at 1% B, 1% B to 5% B in two minutes, 5% B to 90% B in six minutes, hold at 90%B for two minutes, then re-equilibrated at 1% B for two minutes (total analysis time 14 minutes). Electrospray Ionization with an IonKey source was used to introduce the sample into a Synapt G2 HDMS mass spectrometer (Waters), with 2.85 kV ionization, 100C source temperature, 25 V cone potential, 15L/hr cone gas flow and 0.8 bar nanoflow gas pressure. MS measurement was obtained in MSE mode at resolution ~25,000 and scan rate of 5 Hz; low energy scans used 6V collision energy (CE) and high energy scans ramped from 10 to 40 V CE. Metabolite quantitation between samples was accomplished using accurate-mass extracted ion chromatograms of the (M+H)^+^ ion of each metabolite in the Skyline Targeted Quantitation package (daily v 2.6.1, http://skyline.gs.washington.edu/), and relative abundance between conditions was made using area-under-the-curve (AUC) measurements. Retention time and accurate mass used to quantify the native metabolites was verified using spike-in measurements of purchased metabolite standards.

A more quantitatively robust method, UPLC-MS/MS method, was slightly modified from the method above using an Acquity UPLC and Xevo TQ-S triple quadrupole mass spectrometer (Waters corporation), in order to make measurements over a time course of 1 to 4 hours incorporating stable-isotope standards for the amino acids Met and Arg (Figs 6D and S6F). The method was as described above, but used a 2.1 mm x 100 mm BEH column, at a flow rate of 0.4 μl/min and a column temperature of 30°C. The gradient consisted of an initial hold for 1 min at 1% B, followed by a ramp from 1% B to 95% B over 9 minutes, a three minute hold at 95% B, and a four minute re-equilibration at 1% B. The TQ-S source parameters were 3.2 kV ESI, 25 V cone, 150°C source temp, 620°C desolvation temp, cone gas flow of 150 L/hr, and desolvation gas flow of 1000 L/hr, with 15V collision energy.

Finally, for the three hour time point study we utilized the targeted quantitative method described above but we added quantification of ornithine and creatine to the method, as well as stable-isotope internal standards for Ornithine, creatinine, and 5’-methylthioadenosine in order to allow for robust quantification (S6G Fig). Phenylalanine and Spm were quantified in a separate 5x dilution analysis because the samples were too concentrated for these analytes.

### Statistical analyses

Experimental results were analyzed with a Student's t test and graphed using Prism (GraphPad Software, Inc.). Data are expressed as mean ± SD with a p value <0.05 was considered statistically significant.

## Supporting Information

S1 Fig Cellular and transcriptional response to the deprivation of individual amino acids. (**A**) Cell numbers of MCF7 cells under indicated amino acid deprivation for one or two days (n = 3). (**B**) Sub-G1 population of *propidium iodide* (PI) stained MCF7 cells by flow cytometry analysis upon Leu, Gln or Met deprivation at indicated times (n = 3). (**C**) Western blot analysis of phosphorylated eIF2a (S51), S6K1 (T398) and β-tubulin in MCF7 cells after 24 hours of the deprivation of all or indicated individual amino acid. (**D, E, F,**) Heatmap of unsupervised hierarchical clustering of gene expression profiles in MCF7 cells after 24 hours of the deprivation of all or indicated individual amino acid. (**G**) Heatmap of cross-correlations of gene response profiles in the control, all amino acids or individual amino acid deprivation. (**H**) Relative mRNA levels of TXNIP and ARRDC4 by qPCR in MCF7 cells upon the deprivation of all or indicated individual amino acid. (**I**) Relative mRNA levels of p21 (Waf1) and Mdm2 in MCF7 Vector (Vec) and p53 shRNA silenced (shp53) cells upon the deprivation of all or indicated individual amino acid. (**J**) The “R value” projection analysis of common AAR gene signature (AAR-Sig) on Miller tumor dataset (GSE3494). The projection *coefficients* were ranked from high to low. Genetic status of p53, ER and PR and Elston grade of tumors were indicated accordingly.
(PDF)

S2 Fig The methionine-deprived specific transcriptional response. (**A**). The criteria used for identifying the specific genes that are altered by the deprivation of individual amino acid. The table indicated the number of specific probesets that are induced or repressed by the indicated individual amino acid. (**B**) Relative mRNA levels of estrogen receptor (ESR1) in MCF7 cells upon different concentrations (in μM) of methionine treatment for 24 hours. (**C**). Relative levels of the methionine-deprived specific gene signatures (MetDep-Sig) in the CCLE cell lines that grouped by the same primary origin site of cell lines.
(PDF)

S3 Fig The transcriptional response of methionine deprivation is highly similar between MCF7 and PC3 cells. (**A**). Heatmap of the transcriptional change in PC3 (left) and MCF7 (right) cells upon indicated different concentration of methionine treatments for 1 or 2 days. (**B**). Heatmap of unsupervised hierarchical clustering of the baseline gene expression profiles of MCF7 and PC3 cells in control media. (**C**). Relative mRNA levels of genes in MCF7 cells upon methionine deprivation for 24 hours in 0.5% FBS starved condition. (**D**). Western blots analysis of protein level in MCF7 and PC3 cells upon different concentrations (in μM) of methionine for 24 hours in 10% FBS culture medium.
(PDF)

S4 Fig Methionine deprivation reduces the histone methylation. (**A**) A schematic map of the key metabolites and pathways between the methionine, arginine glycine, serine and threonine metabolisms. (**B**) Relative level of DNA methylation in the promoter regions of indicated genes by pyro-sequencing in MCF7 cells after 24 or 48 hours indicated amino acid deprivation. (**C**) Relative level of DNA methylation in the promoter regions of indicated genes by pyro-sequencing in MCF7 cells after 24 hours methionine deprivation. (**D**) Relative level of global DNA methylation by LINE1 assay in MCF7 cells after 24 hours methionine deprivation. (**E**) Heatmap of the methionine-deprived specific transcriptional responsive gene overlapping with the published datasets (GSE17589) of the transcriptional response to the inhibitors of DNA methylation (5-AZA) and histone methylation (DZNep) for 3 days. (**F**). Heatmap of the gene transcriptional response to methionine deprivation (Met-) and 5 μM DZNep treatment (DZN) in MCF7 cells for 24 hours. The probesets were selected with at least 2 fold changes by the treatments relative to the control and arranged by hierarchical clustering. (**G**). Relative levels of methylated histone in the promoter region of indicated genes measured by CHIP-qPCR in MCF7 cells after 48 hours methionine deprivation (HM, 200 μM Methionine; LM, 10 μM methionine; n = 3; #, p < 0.001; *, p < 0.01).
(PDF)

S5 Fig Arginine is required the specific gene response of methionine deprivation. (**A**). Relative expression levels of indicated genes by qPCR in siCon or siSRM MCF7 cells after 24 hours methionine deprivation. (**B**). Relative expression levels of indicated genes by qPCR in MCF7 cells after 24 hours depletion of methionine (-M), or combined with the ODC1 inhibitors POB (200 μM) or DFMO (1mM). (**C**). Relative expression levels of indicated genes by qPCR in MCF7 cells after depletion of methionine (-Met), cystine (-Cys), glutamine (-Gln), co-depletion of methionine and cystine (-Met-Cys) or co-depletion of methionine and glutamine (Met-Gln) for 24 hours. (**D**). Relative expression levels of the indicated genes by qPCR in IMR90 primary cells after depletion of either methionine (-M), or arginine (-R) or both methionine and arginine (-M-R) for 24 hours. (**E**) Heatmap of the methionine-specific transcriptional response (906 probes) in MCF7 cells with deprivation of all amino acids (AA) or methionine (-Met) for 24 hours relative to the control samples. (**F**) Heatmap of the methionine-specific transcriptional response (906 probes) in MCF7 cells after depletion of either methionine (-M), or arginine (-R) or both methionine and arginine (-M-R) for 24 hours.
(PDF)

S6 Fig Arginine/glycine-dependent creatine biosynthesis is required for methionine-deprived specific gene response. (**A**) Relative expression levels of indicated genes by qPCR in MCF7 cells after depletion of methionine (-M), methionine and arginine (-M-R) with or without indicated concentrations of arginase inhibitor nor-NOHA for 24 hours. (**B**) Relative gene expression levels of indicated genes by qPCR in MCF7 cells after depletion of methionine (-Met) with or without indicated concentrations of nitric oxide scavenger cPTIO or nitric oxide synthase inhibitor L-NAME for 24 hours. (**C**) Relative expression levels of indicated genes by qPCR in PC3 cells after depletion of either methionine (-M), arginine (-R), methionine and arginine (-MR), serine (-S), methionine and serine (-MS), glycine (-G), methionine and glycine (-MG), or methionine, serine and glycine (-MSG) for 24 hours. (**D**) Relative expression levels of indicated genes by qPCR in PC3 cells after depletion of either methionine (-M), methionine and arginine (-MR), methionine, threonine and glycine (-MTG), or methionine, threonine, serine and glycine (-MTSG) for 24 hours. (**E**). Western blot analysis of H3K4Me3 and histone H3 (control) in MCF7 or PC3 in indicated deprivation conditions (methionine (M), arginine (R), serine (S), glycine (G) and Threonine (T)) for 24 hours. (**F**) Relative levels of methionine and SAH in MCF7 cells after indicated hours deprivation of methionine (-M), or both methionine and arginine (-MR). (**G**) The absolute or relative levels of metabolites in MCF7 cells after 3 hours deprivation of methionine (-M), arginine (-R), or both methionine and arginine (-MR) (n = 3; #, p < 0.005; *, p < 0.05; ##, p < 0.005). (**H**). Western blot analysis of AGAT expression in shRNA scramble (shScr), silenced AGAT (shAGAT) or GAMT (shGAMT) MCF7 cells after 24 hours methionine deprivation.
(PDF)

S7 Fig The ornithine-mediated signaling is required for the complete induction of the methionine-deprived specific gene response. (**A**). Relative expression levels of indicated genes by qPCR in MCF7 cells after 24 hours depletion of either methionine (-M), methionine and arginine (-MR) with or without the addition of 0.4 mM ornithine (ORN). (B) Relative expression levels of indicated genes by qPCR in MCF7 cells after 24 hours depletion of either methionine (-M), methionine and arginine (-MR) with or without the addition of 0.4 mM creatine (Cr).
(PDF)

S1 Text Supplemental Materials and Methods. (DOCX)

S1 Table The probeset list of common amino acid response (778 probesets). (XLSX)

S2 Table The probeset list of individual amino acid specific response. (XLSX)

S3 Table The probeset list of methionine deprived specific response. (XLSX)

S4 Table TRANSFEC analysis of the methionine-deprived specific response genes using Gather. (XLSX)

S5 Table The primer information for qPCR, CHIP-qPCR, and pyrosequencing. (XLSX)

S6 Table shRNA plasmids information. (XLSX)

S1 File Supplemental metabolomics skyline methods. (ZIP)
