# Supplementary figures and images for: Comprehensive Profiling of Amino Acid Response Uncovers Unique Methionine-Deprived Response Dependent on Intact Creatine Biosynthesis
Source: PLoS Genet. 2015 Apr 7;11(4):e1005158. doi: 10.1371/journal.pgen.1005158 (PMC4388453; doi:10.1371/journal.pgen.1005158)

Fig S1

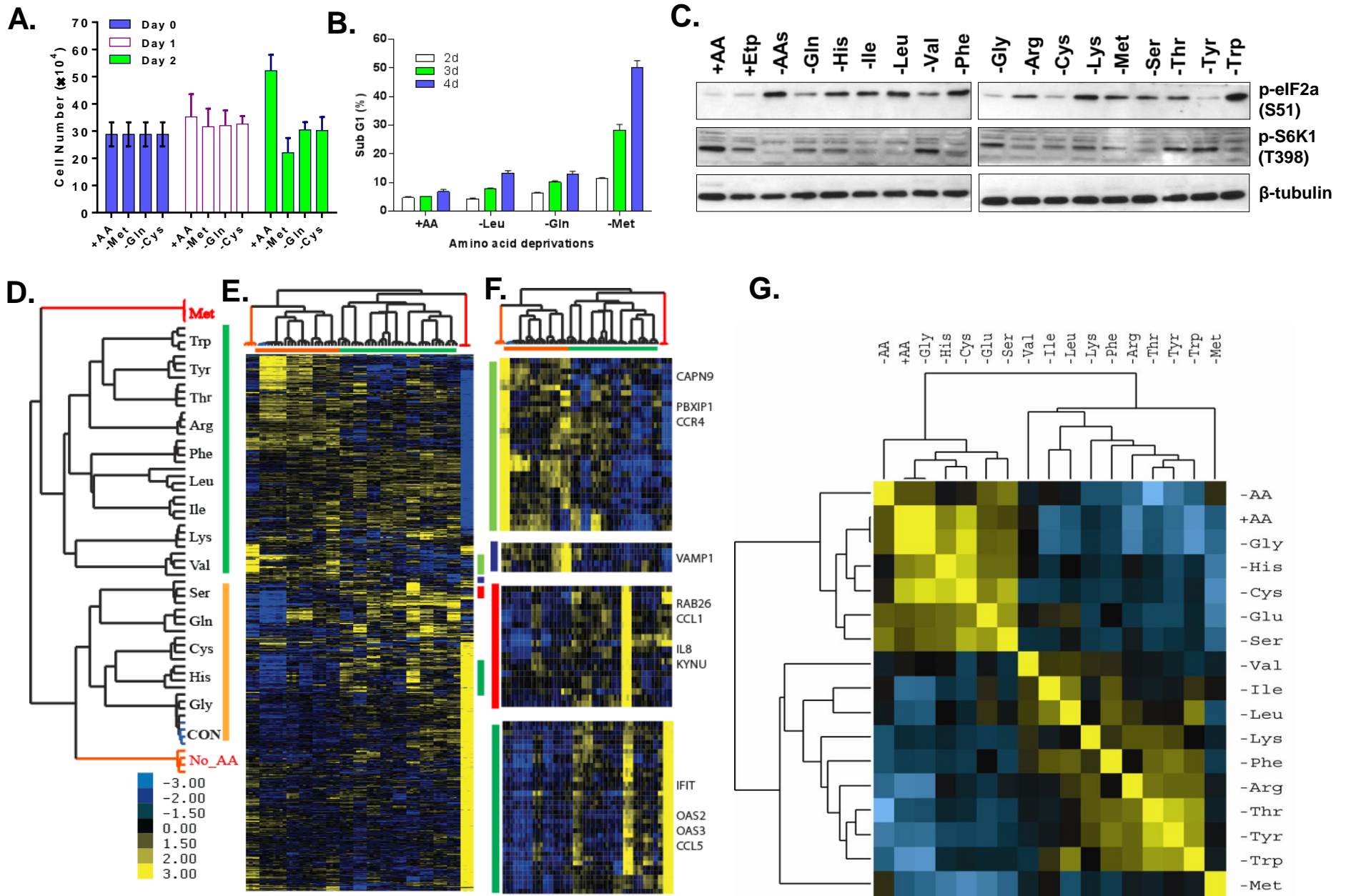

Fig S1 cont'

H.

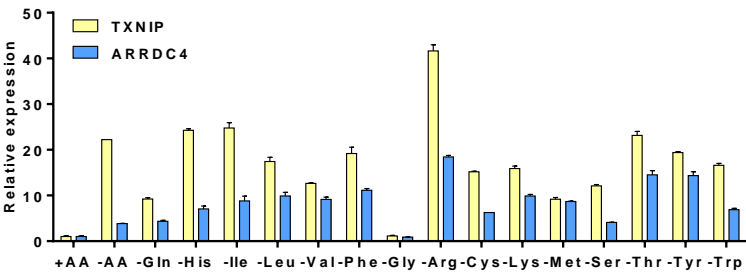

I.

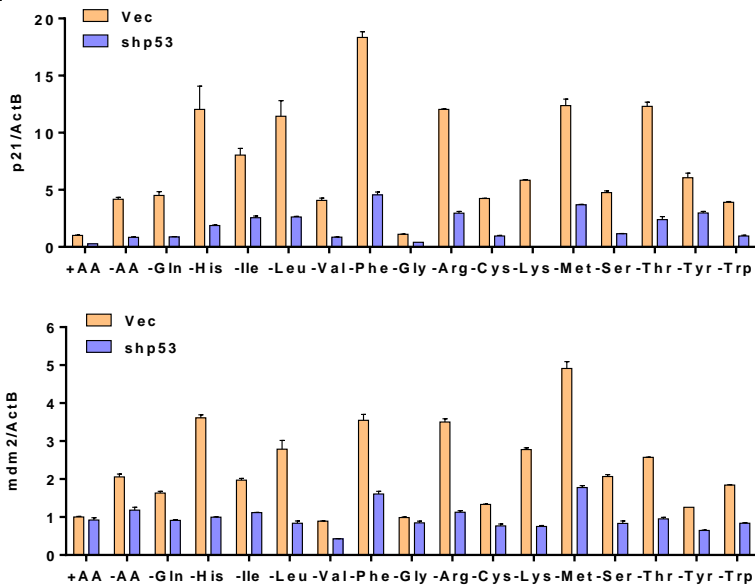

J. Common AAR-Sig projection

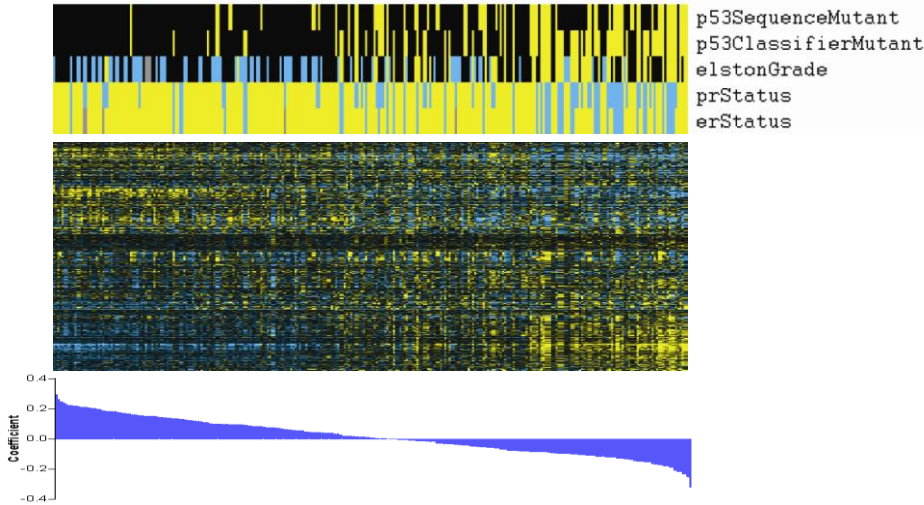

Supplement: S1 Fig — (A) Cell numbers of MCF7 cells under indicated amino acid deprivation for one or two days (n = 3). (B) Sub-G1 population of propidium iodide (PI) stained MCF7 cells by flow cytometry analysis upon Leu, Gln or Met deprivation at indicated times (n = 3). (C) Western blot analysis of phosphorylated eIF2a (S51), S6K1 (T398) and β-tubulin in MCF7 cells after 24 hours of the deprivation of all or indicated individual amino acid. (D, E, F,) Heatmap of unsupervised hierarchical clustering of gene expression profiles in MCF7 cells after 24 hours of the deprivation of all or indicated individual amino acid. (G) Heatmap of cross-correlations of gene response profiles in the control, all amino acids or individual amino acid deprivation. (H) Relative mRNA levels of TXNIP and ARRDC4 by qPCR in MCF7 cells upon the deprivation of all or indicated individual amino acid. (I) Relative mRNA levels of p21 (Waf1) and Mdm2 in MCF7 Vector (Vec) and p53 shRNA silenced (shp53) cells upon the deprivation of all or indicated individual amino acid. (J) The “R value” projection analysis of common AAR gene signature (AAR-Sig) on Miller tumor dataset (GSE3494). The projection coefficients were ranked from high to low. Genetic status of p53, ER and PR and Elston grade of tumors were indicated accordingly. (PDF) [file pgen.1005158.s001.pdf]

Fig S3

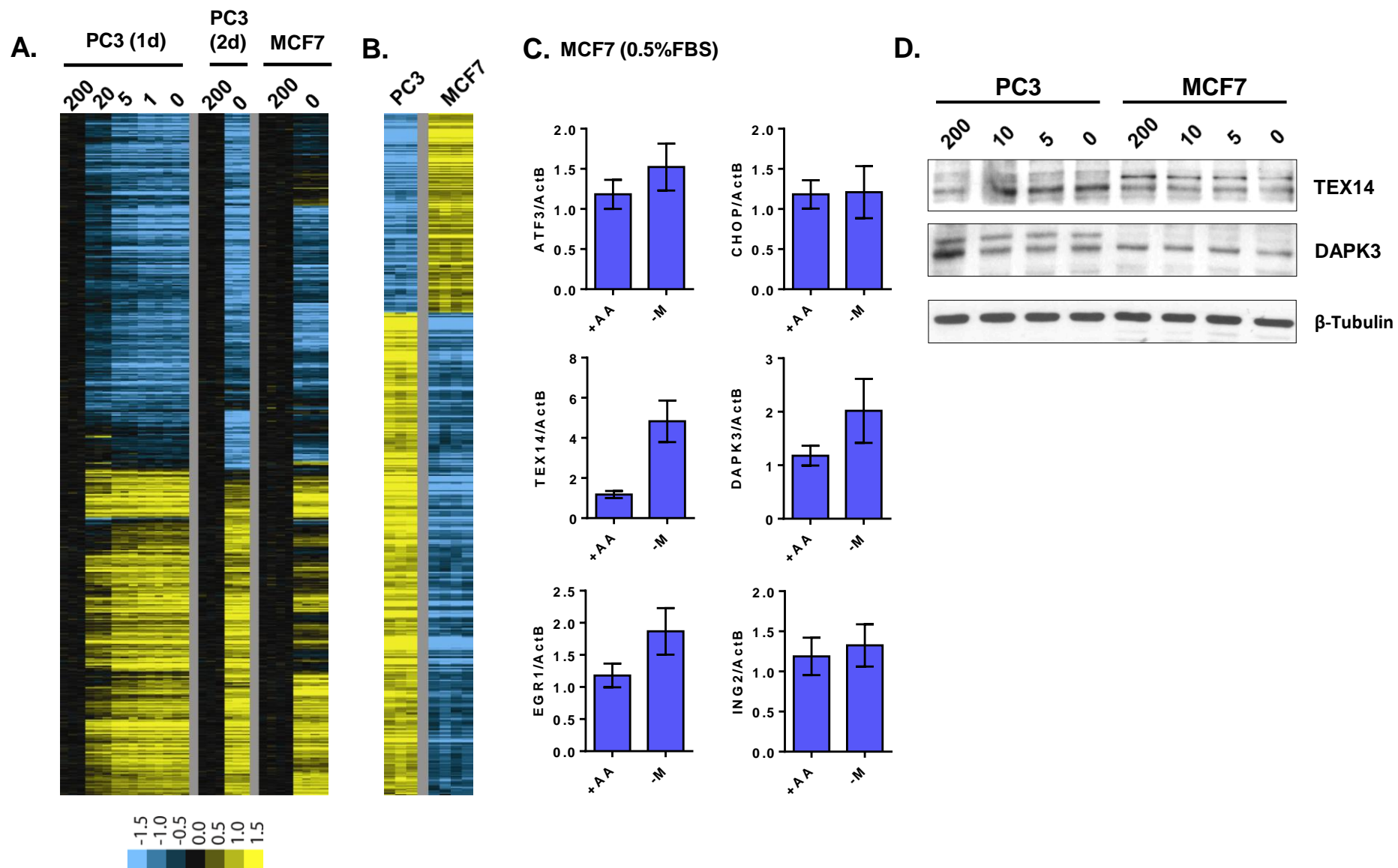

Supplement: S3 Fig — (A). Heatmap of the transcriptional change in PC3 (left) and MCF7 (right) cells upon indicated different concentration of methionine treatments for 1 or 2 days. (B). Heatmap of unsupervised hierarchical clustering of the baseline gene expression profiles of MCF7 and PC3 cells in control media. (C). Relative mRNA levels of genes in MCF7 cells upon methionine deprivation for 24 hours in 0.5% FBS starved condition. (D). Western blots analysis of protein level in MCF7 and PC3 cells upon different concentrations (in μM) of methionine for 24 hours in 10% FBS culture medium. (PDF) [file pgen.1005158.s003.pdf]

**A.**

**B.**

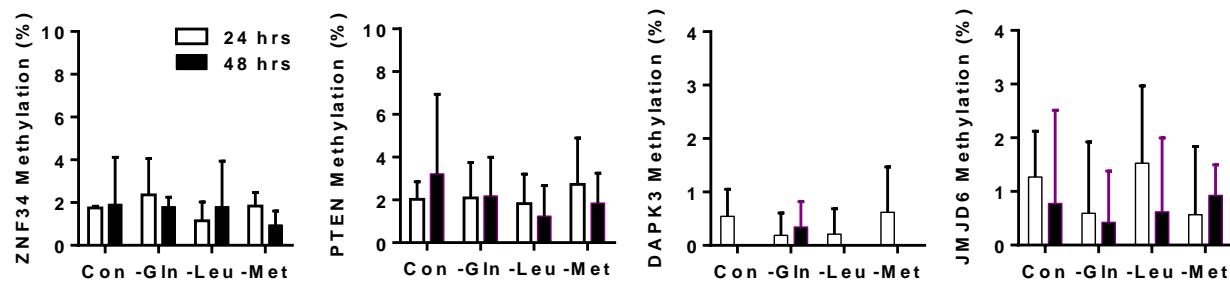

**C.**

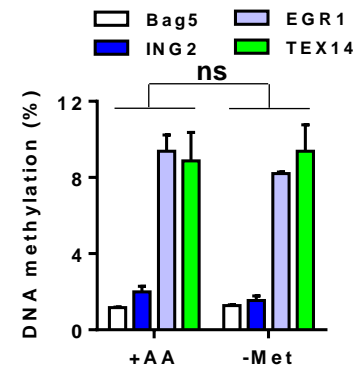

**D.**

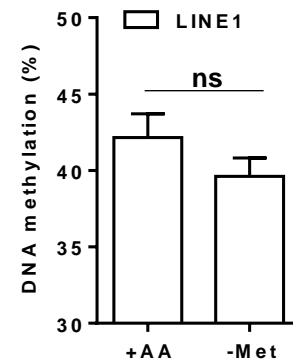

Fig S4 cont'

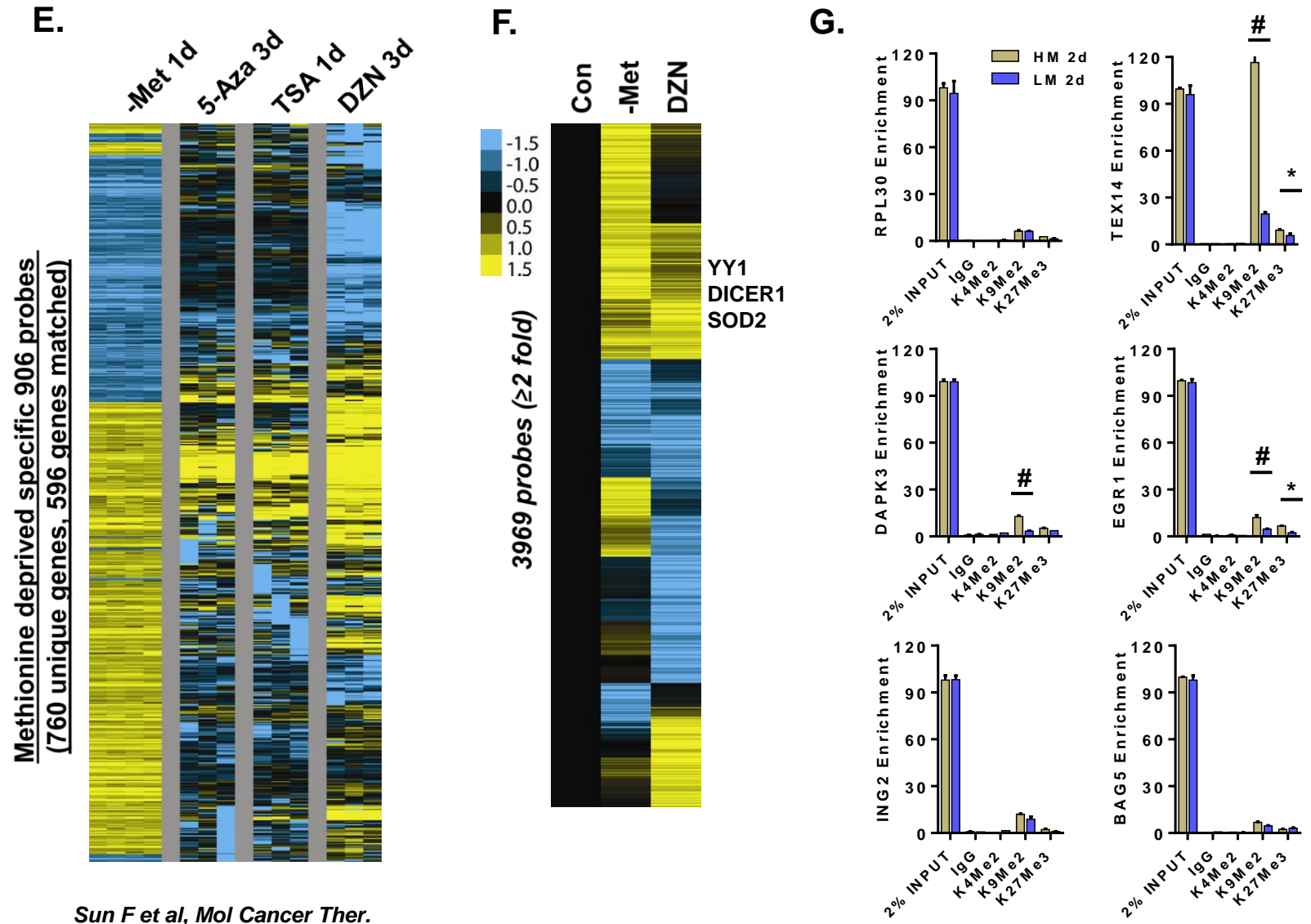

Sun F et al, Mol Cancer Ther.  
2009 Dec;8(12):3191-202.

Supplement: S4 Fig — (A) A schematic map of the key metabolites and pathways between the methionine, arginine glycine, serine and threonine metabolisms. (B) Relative level of DNA methylation in the promoter regions of indicated genes by pyro-sequencing in MCF7 cells after 24 or 48 hours indicated amino acid deprivation. (C) Relative level of DNA methylation in the promoter regions of indicated genes by pyro-sequencing in MCF7 cells after 24 hours methionine deprivation. (D) Relative level of global DNA methylation by LINE1 assay in MCF7 cells after 24 hours methionine deprivation. (E) Heatmap of the methionine-deprived specific transcriptional responsive gene overlapping with the published datasets (GSE17589) of the transcriptional response to the inhibitors of DNA methylation (5-AZA) and histone methylation (DZNep) for 3 days. (F). Heatmap of the gene transcriptional response to methionine deprivation (Met-) and 5 μM DZNep treatment (DZN) in MCF7 cells for 24 hours. The probesets were selected with at least 2 fold changes by the treatments relative to the control and arranged by hierarchical clustering. (G). Relative levels of methylated histone in the promoter region of indicated genes measured by CHIP-qPCR in MCF7 cells after 48 hours methionine deprivation (HM, 200 μM Methionine; LM, 10 μM methionine; n = 3; #, p < 0.001; *, p < 0.01). (PDF) [file pgen.1005158.s004.pdf]

**Fig S5**

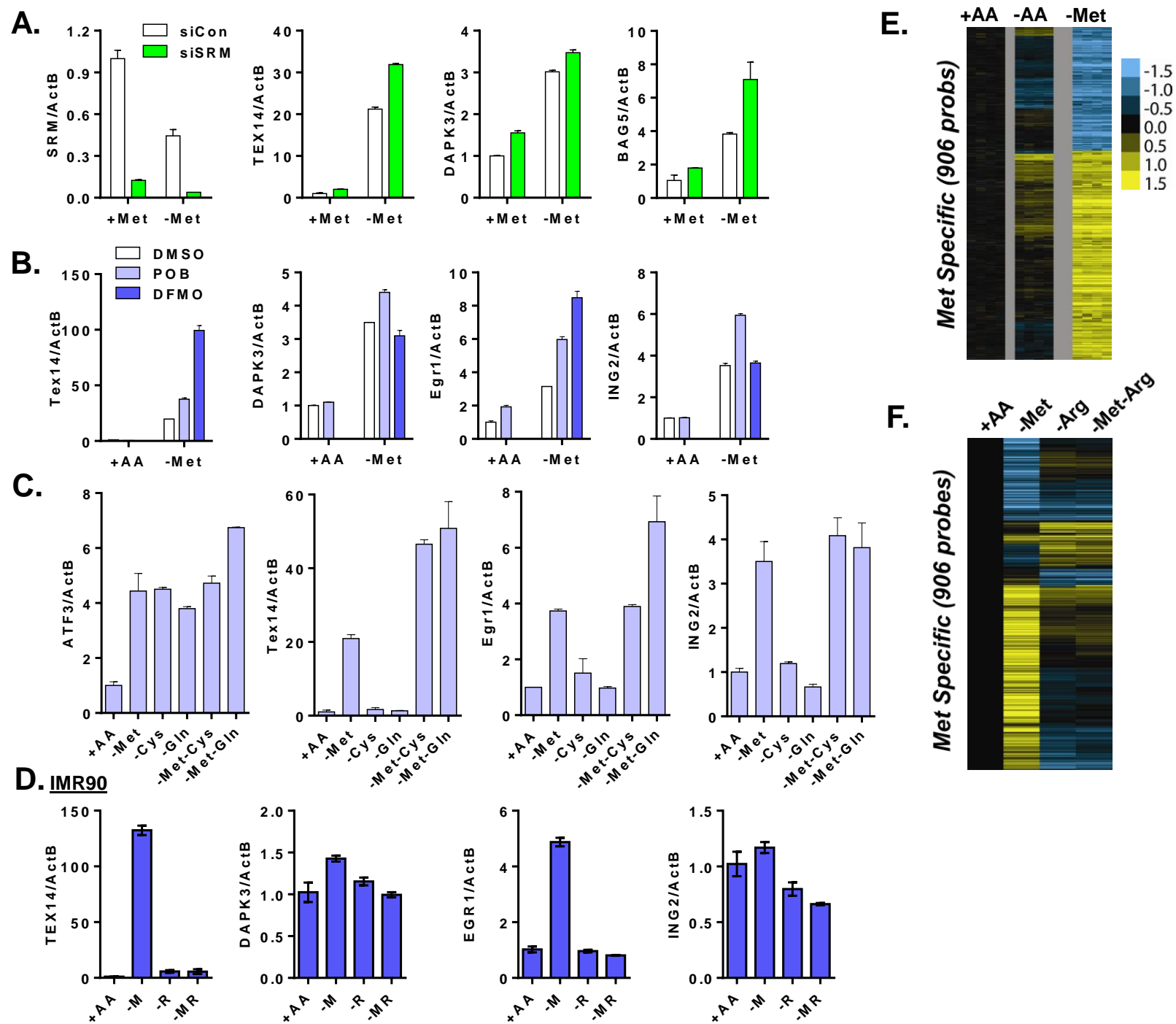

Supplement: S5 Fig — (A). Relative expression levels of indicated genes by qPCR in siCon or siSRM MCF7 cells after 24 hours methionine deprivation. (B). Relative expression levels of indicated genes by qPCR in MCF7 cells after 24 hours depletion of methionine (-M), or combined with the ODC1 inhibitors POB (200 μM) or DFMO (1mM). (C). Relative expression levels of indicated genes by qPCR in MCF7 cells after depletion of methionine (-Met), cystine (-Cys), glutamine (-Gln), co-depletion of methionine and cystine (-Met-Cys) or co-depletion of methionine and glutamine (Met-Gln) for 24 hours. (D). Relative expression levels of the indicated genes by qPCR in IMR90 primary cells after depletion of either methionine (-M), or arginine (-R) or both methionine and arginine (-M-R) for 24 hours. (E) Heatmap of the methionine-specific transcriptional response (906 probes) in MCF7 cells with deprivation of all amino acids (AA) or methionine (-Met) for 24 hours relative to the control samples. (F) Heatmap of the methionine-specific transcriptional response (906 probes) in MCF7 cells after depletion of either methionine (-M), or arginine (-R) or both methionine and arginine (-M-R) for 24 hours. (PDF) [file pgen.1005158.s005.pdf]

Fig S6

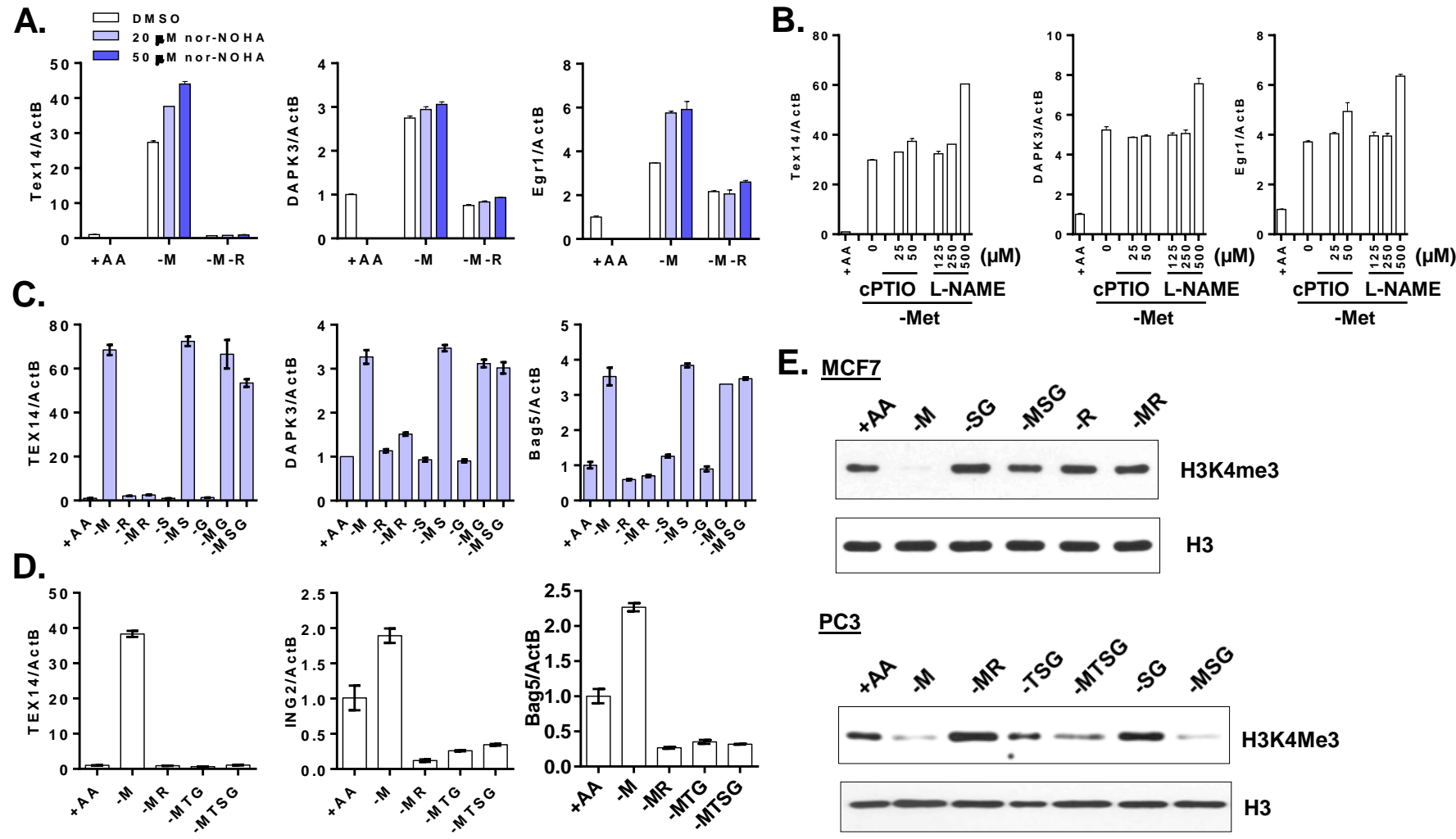

Fig S6 cont'

F.

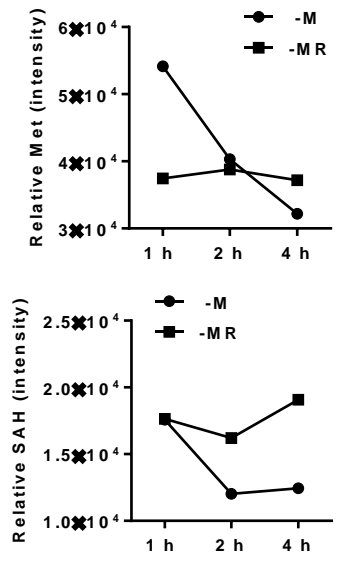

G.

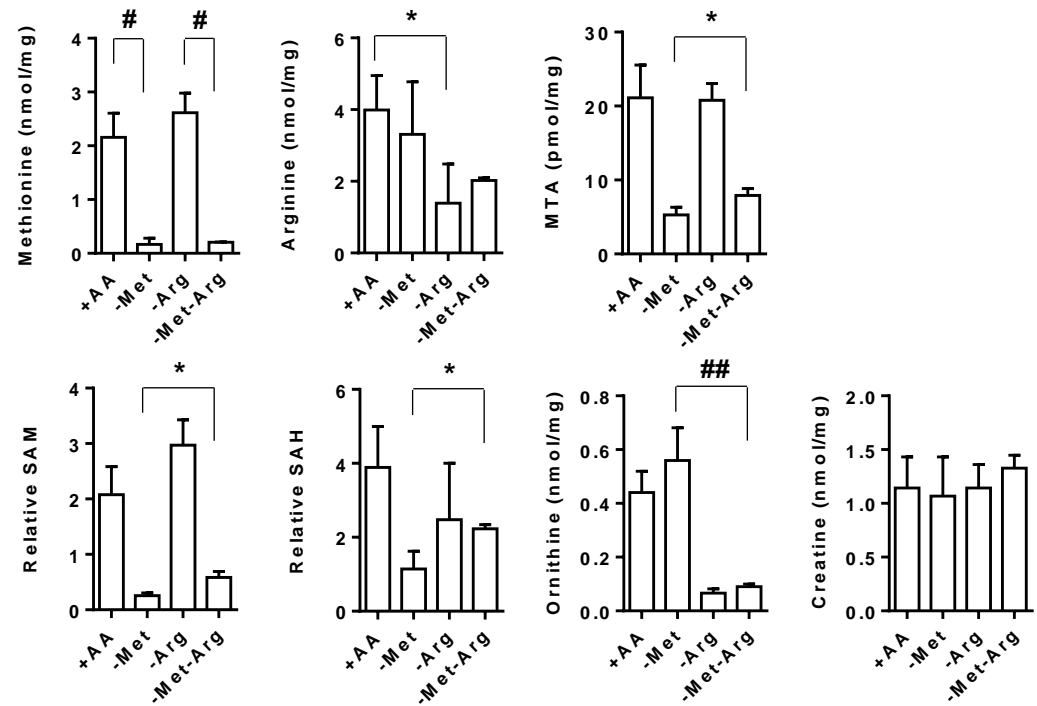

H.

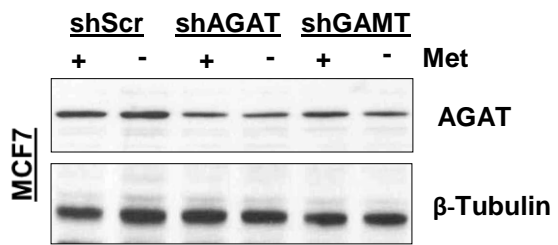

Supplement: S6 Fig — (A) Relative expression levels of indicated genes by qPCR in MCF7 cells after depletion of methionine (-M), methionine and arginine (-M-R) with or without indicated concentrations of arginase inhibitor nor-NOHA for 24 hours. (B) Relative gene expression levels of indicated genes by qPCR in MCF7 cells after depletion of methionine (-Met) with or without indicated concentrations of nitric oxide scavenger cPTIO or nitric oxide synthase inhibitor L-NAME for 24 hours. (C) Relative expression levels of indicated genes by qPCR in PC3 cells after depletion of either methionine (-M), arginine (-R), methionine and arginine (-MR), serine (-S), methionine and serine (-MS), glycine (-G), methionine and glycine (-MG), or methionine, serine and glycine (-MSG) for 24 hours. (D) Relative expression levels of indicated genes by qPCR in PC3 cells after depletion of either methionine (-M), methionine and arginine (-MR), methionine, threonine and glycine (-MTG), or methionine, threonine, serine and glycine (-MTSG) for 24 hours. (E). Western blot analysis of H3K4Me3 and histone H3 (control) in MCF7 or PC3 in indicated deprivation conditions (methionine (M), arginine (R), serine (S), glycine (G) and Threonine (T)) for 24 hours. (F) Relative levels of methionine and SAH in MCF7 cells after indicated hours deprivation of methionine (-M), or both methionine and arginine (-MR). (G) The absolute or relative levels of metabolites in MCF7 cells after 3 hours deprivation of methionine (-M), arginine (-R), or both methionine and arginine (-MR) (n = 3; #, p < 0.005; *, p < 0.05; ##, p < 0.005). (H). Western blot analysis of AGAT expression in shRNA scramble (shScr), silenced AGAT (shAGAT) or GAMT (shGAMT) MCF7 cells after 24 hours methionine deprivation. (PDF) [file pgen.1005158.s006.pdf]

Fig S7.

**A. Orn: 0.4 mM ornithine**

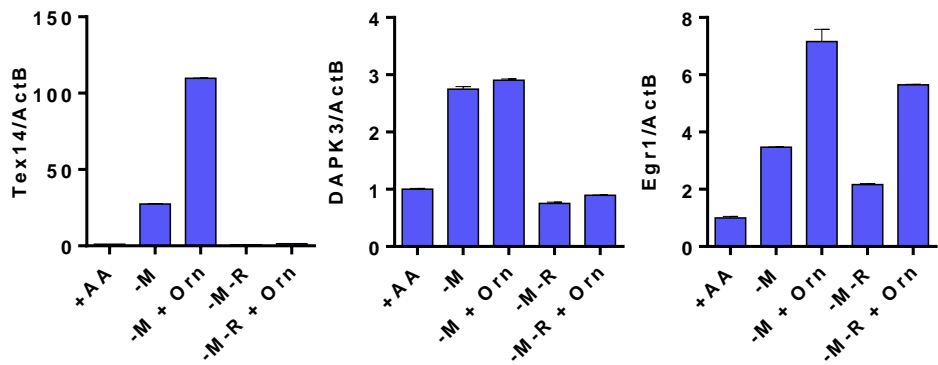

**B. Cr: 0.4 mM Creatine**

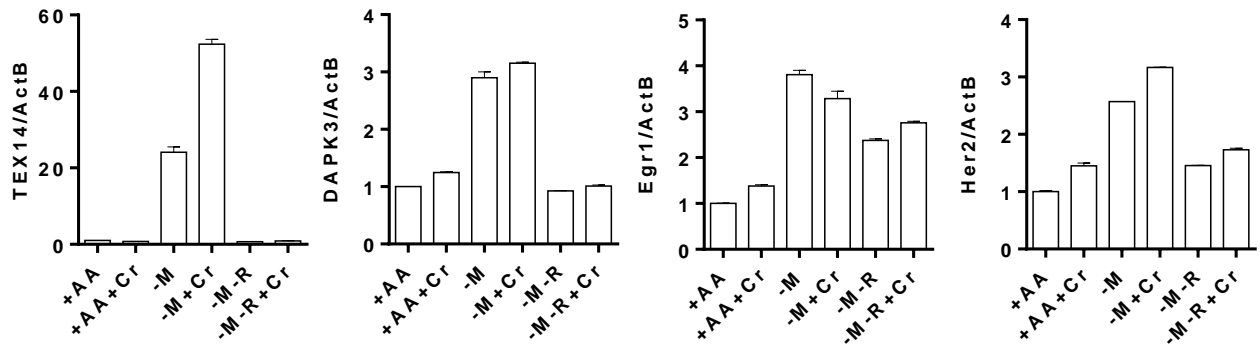

Supplement: S7 Fig — (A). Relative expression levels of indicated genes by qPCR in MCF7 cells after 24 hours depletion of either methionine (-M), methionine and arginine (-MR) with or without the addition of 0.4 mM ornithine (ORN). (B) Relative expression levels of indicated genes by qPCR in MCF7 cells after 24 hours depletion of either methionine (-M), methionine and arginine (-MR) with or without the addition of 0.4 mM creatine (Cr). (PDF) [file pgen.1005158.s007.pdf]
