# Supplementary material for: Comprehensive Profiling of Amino Acid Response Uncovers Unique Methionine-Deprived Response Dependent on Intact Creatine Biosynthesis
Source: PLoS Genet. 2015 Apr 7;11(4):e1005158. doi: 10.1371/journal.pgen.1005158 (PMC4388453; doi:10.1371/journal.pgen.1005158)

# Fig S2

**A.**

**Criteria for the specific gene response:**

$\Delta Log_2(\$i) >= 0.8$; $\Delta Log_2(\$j) < 0.5$, if the level of a probe is induced;

$\Delta Log_2(\$i) <= -0.8$; $\Delta Log_2(\$j) > -0.5$, if the level of a probe is repressed;

$i : The probe level of a targeted amino acid deprivation;

$j : The probe level of any other amino acid deprivation.

| Treatment | Specificity | | |
|---|---|---|---|
| | All | Up | Down |
| +AAs | 0 | 0 | 0 |
| -Gln | 3 | 1 | 2 |
| -His | 2 | 1 | 1 |
| -Ile | 12 | 2 | 10 |
| -Leu | 0 | 0 | 0 |
| -Val | 3 | 0 | 3 |
| -Phe | 14 | 1 | 13 |
| -Gly | 0 | 0 | 0 |
| -Arg | 24 | 9 | 15 |
| -Cys | 2 | 1 | 1 |
| -Lys | 39 | 13 | 26 |
| -Met | 906 | 568 | 338 |
| -Ser | 10 | 10 | 0 |
| -Thr | 21 | 12 | 9 |
| -Tyr | 6 | 2 | 4 |
| -Trp | 19 | 4 | 15 |

**B.**

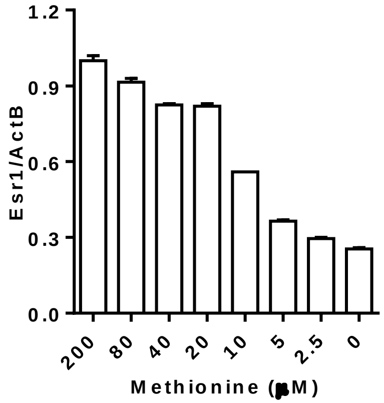


**C.**

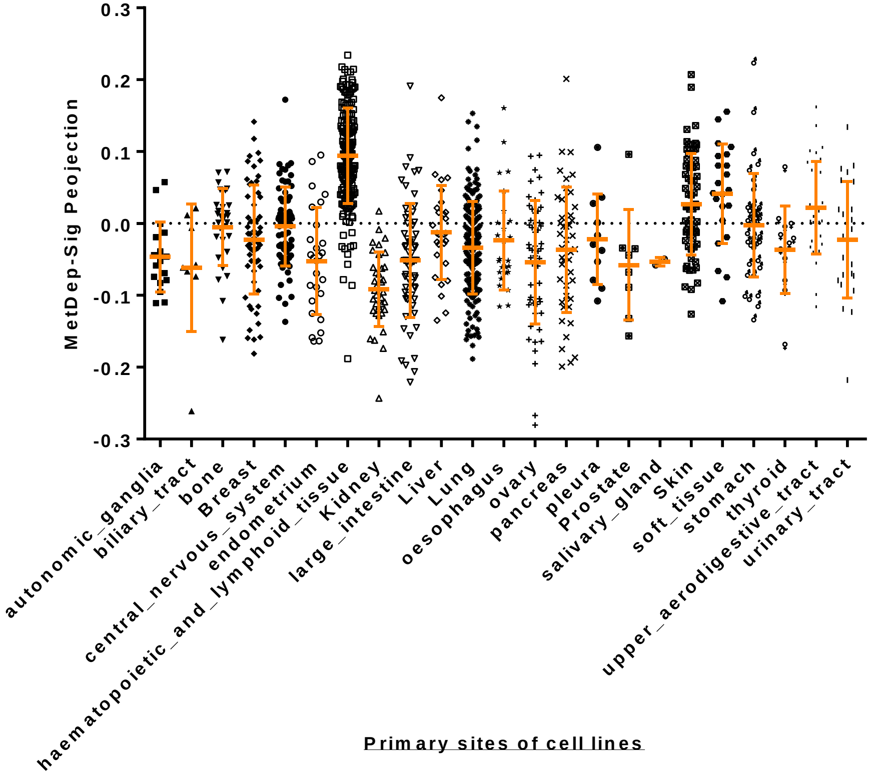

Supplement: S2 Fig — (A). The criteria used for identifying the specific genes that are altered by the deprivation of individual amino acid. The table indicated the number of specific probesets that are induced or repressed by the indicated individual amino acid. (B) Relative mRNA levels of estrogen receptor (ESR1) in MCF7 cells upon different concentrations (in μM) of methionine treatment for 24 hours. (C). Relative levels of the methionine-deprived specific gene signatures (MetDep-Sig) in the CCLE cell lines that grouped by the same primary origin site of cell lines. (PDF) [file pgen.1005158.s002.pdf]
