# Supplementary material for: Comprehensive Profiling of Amino Acid Response Uncovers Unique Methionine-Deprived Response Dependent on Intact Creatine Biosynthesis
Source: PLoS Genet. 2015 Apr 7;11(4):e1005158. doi: 10.1371/journal.pgen.1005158 (PMC4388453; doi:10.1371/journal.pgen.1005158)
Supplement: S1 Text — (DOCX) [file pgen.1005158.s008.docx]

**Supplemental Materials and Methods**

### Materials

Chemical materials were obtained from the following sources: Cell culture grade amino acids, SAM, SAH, Hcy, MTA, ornithine, creatine and GAA from Sigma; DFMO from Santa cruz and POB from EMD; DZNep, UNC0638, nor-NOHA, L-NAME and c-PTIO from Cayman chemical. The shRNA plasmids targeting AGAT and GAMT genes were from Sigma and listed in S6 Table.

**Western blot analysis**

Cells under the indicated conditions were washed with cold PBS and lysed with NP40 buffer (50 mmol/L Tris pH 8.0, 150 mmol/L NaCl, 1% NP-40) supplemented with protease inhibitor and phosphatase inhibitor cocktail (Roche) for the extraction of total protein. Histone proteins were isolated by EpiQuik Total Histone Extraction Kit (Epigentek). The concentrations were determined with BCA protein assay. Equal amounts of protein were loaded for the immunoblot analyses. Primary antibodies of S6K1 (whole and pho-T398, cell signaling), eIF2a (pho-Ser51, cell signaling), Histone and histone methylation antibodies (Cell signaling and Active Motif) and β-tubulin (Sigma) were applied following the manufacturers' protocols. The signal was detected by the ECL plus Western blotting detection system (Amersham).

**LC-MS/MS Methods**

The Skyline software document that contains precursor and product ion information for all metabolites measured in this study, and their internal standards, has been provided as a supplementary file to the manuscript: S1 File-Metabolomics_Skyline_methods.sky.zip.
